# Supplementary material for: Microbial metabolites tune amygdala neuronal hyperexcitability and anxiety-linked behaviors
Source: EMBO Mol Med. 2025 Feb 5;17(2):249–64. doi: 10.1038/s44321-024-00179-y (PMC11821874; doi:10.1038/s44321-024-00179-y)
Supplement: Supplementary file 1 — Appendix [file 44321_2024_179_MOESM1_ESM.pdf]

Appendix Table of Content

1. **Appendix Figure S1** **Pg 2**
2. **Appendix Figure S2** **Pg 3**
3. **Appendix Figure S3** **Pg 5**
4. **Appendix Figure S4** **Pg 7**
5. **Appendix Figure S5** **Pg 8**
6. **Appendix Table S1** **Pg 10**
7. **Appendix Table S2** **Pg 11**

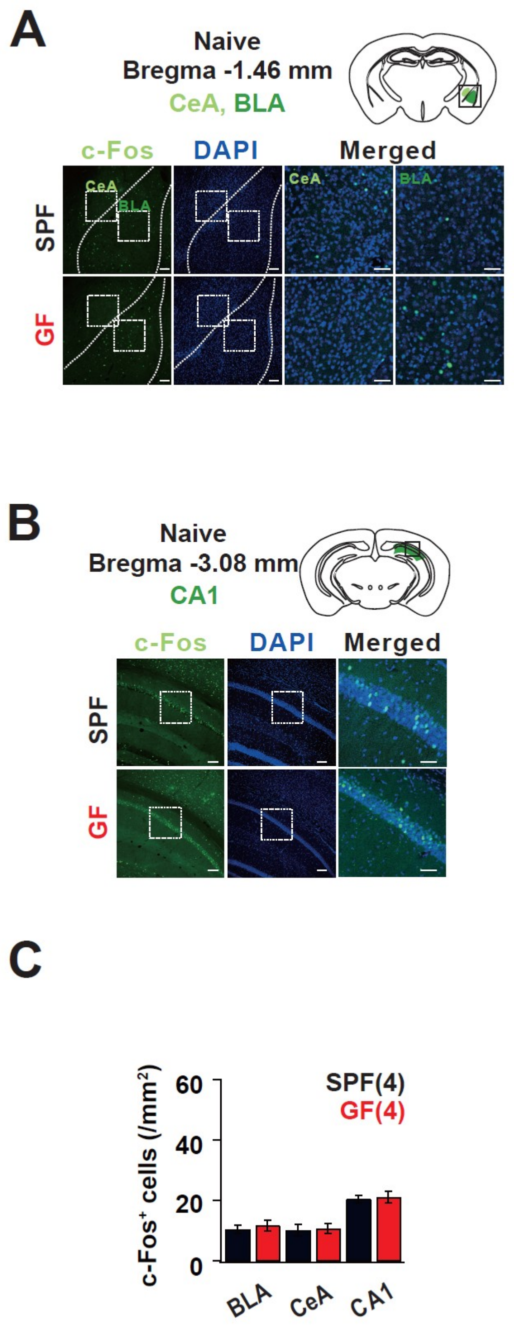

Appendix Figure S1. Expression of c-Fos in neurons of the amygdala and CA1 region in naïve mice.

(A-B) Activity mapping of the amygdala and CA1 using c-Fos staining in naïve SPF control and GF mice. A magnified image of c-Fos immunoreactivity corresponding to the inset in the fluorescence image is shown in "Merged". Representative images of c-Fos positive cells in various brain regions. Schematics were drawn based on the Allen Mouse Brain Atlas. (C) Quantification of the number of c-Fos-positive (+) cells in the naïve SPF control and GF mouse groups. BLA; basolateral amygdala, CeA; central amygdala. Scale bars: 100 µm in (left) and 50 µm in (right).

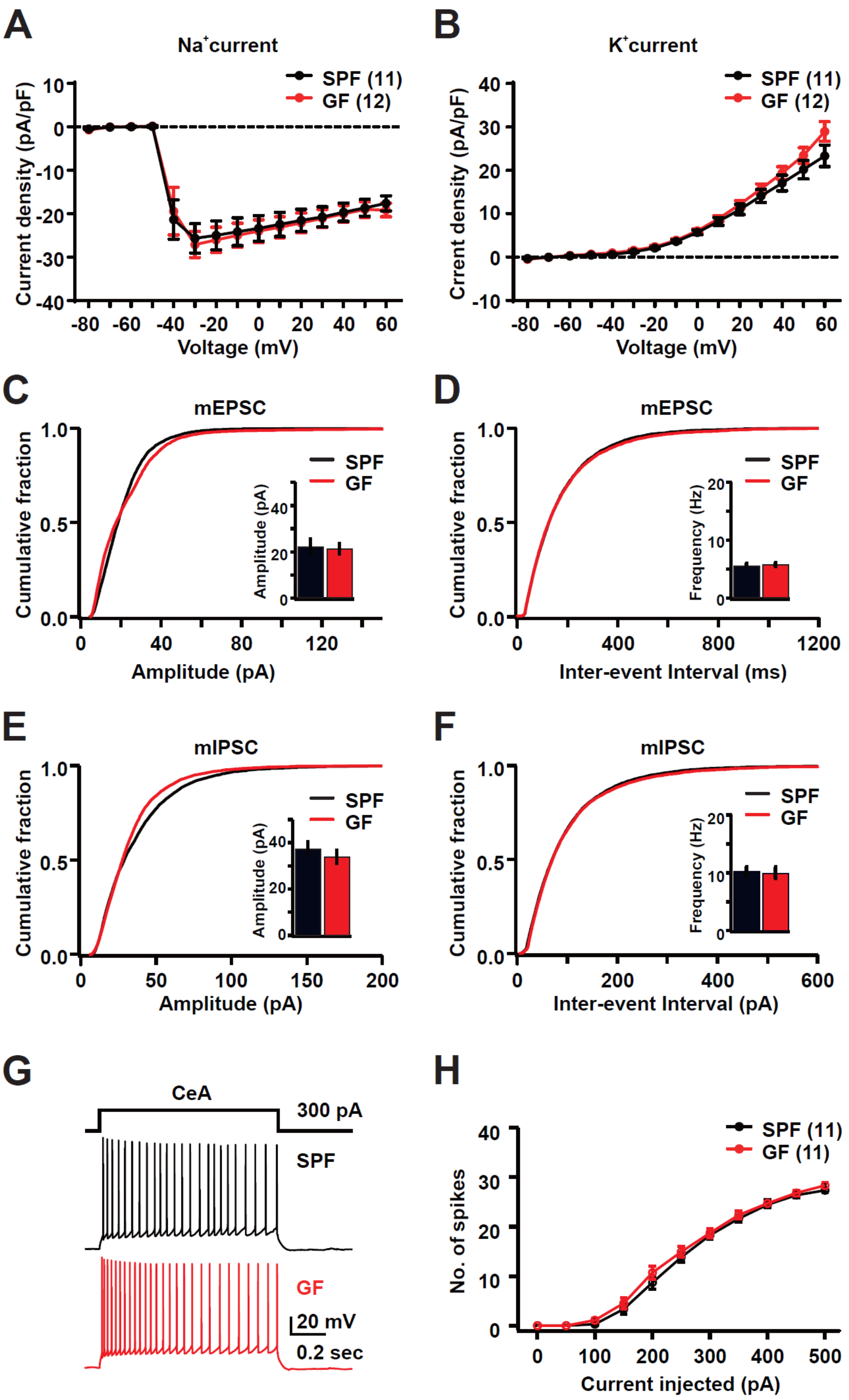
A
Na+ current
SPF (11)
GF (12)
Current density (pA/pF)
Voltage (mV)
B
K+ current
SPF (11)
GF (12)
Crent density (pA/pF)
Voltage (mV)
C
mEPSC
SPF
GF
Amplitude (pA)
Cumulative fraction
Amplitude (pA)
D
mEPSC
SPF
GF
Frequency (Hz)
Cumulative fraction
Inter-event Interval (ms)
E
mIPSC
SPF
GF
Amplitude (pA)
Cumulative fraction
Amplitude (pA)
F
mIPSC
SPF
GF
Frequency (Hz)
Cumulative fraction
Inter-event Interval (pA)
G
CeA
300 pA
SPF
GF
20 mV
0.2 sec
H
SPF (11)
GF (11)
No. of spikes
Current injected (pA)

Appendix Figure S2. No detectable change in voltage-gated, sodium and potassium channels in the principal neurons in the BLA of GF mice compared to SPF.

(A) Quantification of the current density of different voltage-gated Na^+ currents (A) and K^+ currents. (B) Induced by voltage steps from -80 mV to +60 mV in BLA neurons from SPF and GF mice. (C-F) Analysis of mEPSCs and mIPSCs recorded from basolateral amygdala (BLA) neurons in SPF and GF mice. Data show cumulative probability distribution of mEPSC and mIPSC amplitude and inter-event interval, and the quantification of amplitudes and frequencies recorded from BLA neurons in SPF and GF mice. (G) Representative traces of spike trains induced in central amygdala neurons from SPF and GF mice in response to current injections (400pA). (H) The number of spikes induced by a 1 s current step ranging from 0 to 500 pA with a 50 pA increment in central amygdala neurons from SPF and GF mice (SPF: n = 11, GF: n = 11, N = 3 mice per group).

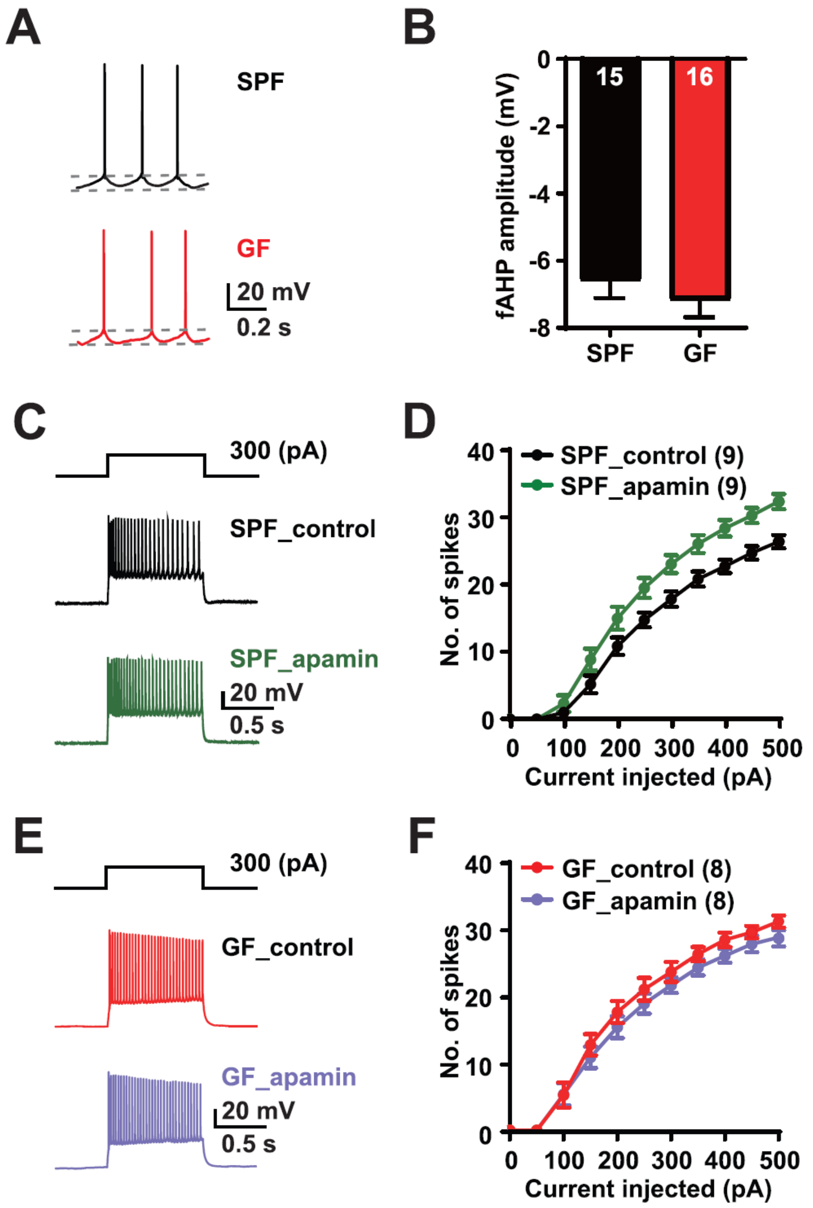

Appendix Figure S3. No significant change in fAHP and a lack of apamin-induced change in neuronal excitability in GF BLA neurons.

(A) Representative traces of spike trains recorded in the principal BLA neurons from SPF and GF mice. The dashed line indicates fAHP amplitude measurements. Scale bar: 20 mV, 0.5 s. (B) Quantification of fAHP amplitude recorded from BLA neurons in SPF and GF neurons. (C) Representative traces of spike trains elicited in GF BLA neurons before (black) and after (green) apamin application. Scale bar 20 mV, 0.5 s. (D) Quantification of the number of spikes elicited by a 1 s current step ranging from 0 to 500 pA in BLA neurons from SPF mice before and after apamin treatment (100 nM). n = 9 cells, N = 3 mice. (E) Representative spike train traces induced in GF

BLA neurons before (red) and after (purple) apamin application (100 nM). Scale bar 20 mV, 0.5 s. (F) Quantification of spike counts induced by a 1 s current step ranging from 0 to 500 pA in BLA neurons from GF mice before and after apamin treatment (100 nM). n = 9 cells, N = 3 mice, n = 8 cells, N = 3 mice. Data are expressed as mean ± SEM. Student's t-test. All mice were aged 10 to 14 weeks old at the time of testing.

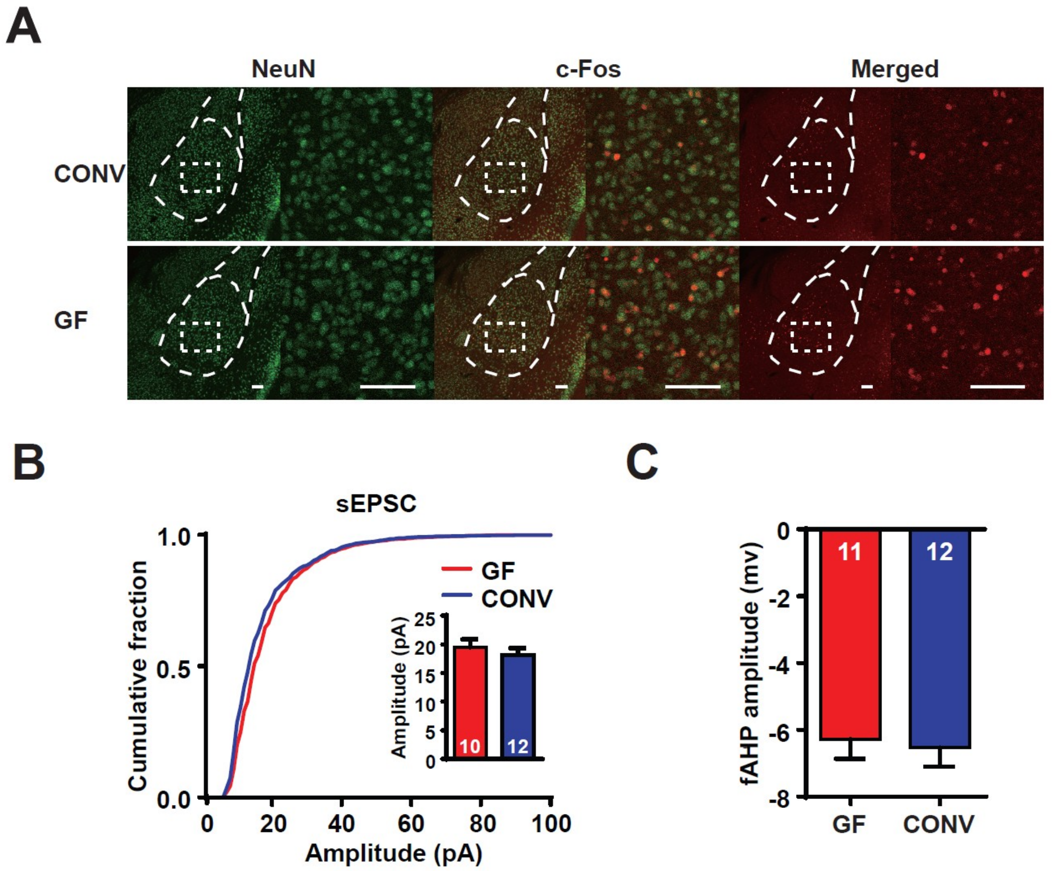

Appendix Figure S4. Normalized BLA hyperactivity in CONV mice.

(A) Representative coronal sections stained for c-Fos and NeuN after the EZM. The dashed line indicates the location of the BLA. The right image in each panel is a magnification of the BLA portion shown in the left image. (Scare bar: 0.1 mm, 100 mm). (B) Cumulative probability distribution of sEPSC amplitude and quantification of the mean sEPSC amplitude. GF n = 10 cells N = 4 mice, GF n = 12 cells N = 4 mice. (C) Quantification of fAHP amplitude recorded from BLA neurons of GF and CONV mice. **p < 0.01. Data are expressed as mean ± SEM. Student's t-test. All mice were aged 10 to 12 weeks old at the time of testing.

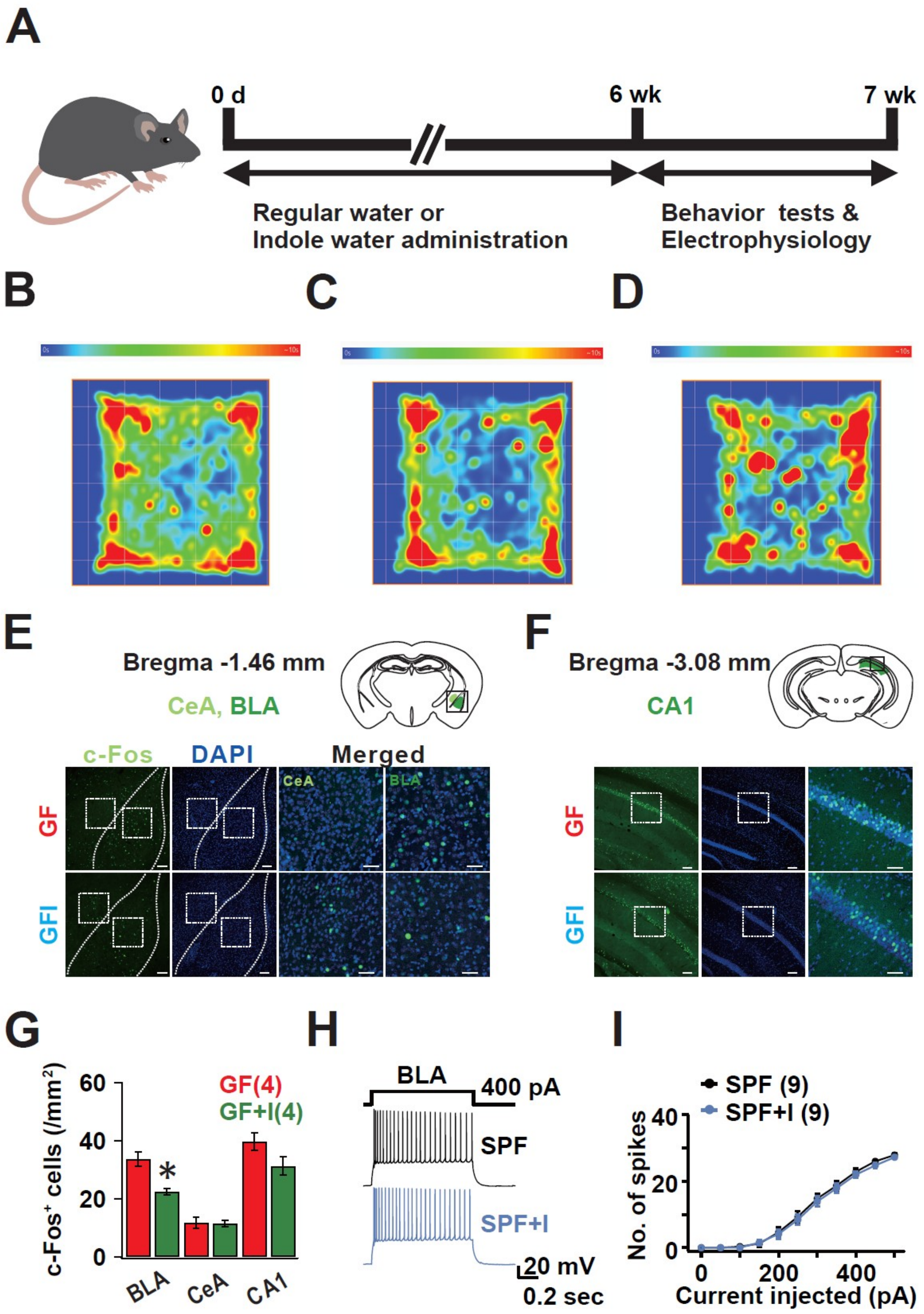

Appendix Figure S5. Indole reduces anxiety and BLA hyperactivity in germ-free mice.

(A) Experimental design for the oral administration of indole to GF mice: Male GF mice were randomly assigned to receive either normal drinking water or water supplemented with 200 μM indole for a period of 6 weeks. (B-D) Representative heat map images showing the time spent in

different areas by SPF, GF, and GF+I mice [red (10 s or more) > yellow > green > blue (0 s)]. N = 10 (SPF), 8 (GF), 8 (GF+I). (E-F) c-Fos staining was used to map neuronal activity in the amygdala and CA1 region of germ-free (GF) mice after the elevated zero maze (EZM) experiment. The most significant differences in c-Fos expression were observed in the basolateral amygdala (BLA). A magnified view of the c-Fos immunoreactivity, corresponding to the inset in the fluorescence image, is shown the merged panel. Scale bars: 100 μm in (left) and 50 μm in (right). (G) Quantitative analysis of c-Fos-positive (+) cells in GF mice and indole-treated GF mice. (H) Representative traces of spike trains induced in BLA pyramidal neurons in SPF and SPF+I mice in response to current injections (400 pA). Scale bar 20 mV, 0.2 s. (I) The number of spikes induced by a 1 s current step ranging from 0 to 500 pA with a 50 pA increment in BLA pyramidal neurons in SPF and SPF+I mice (SPF: n = 9, SPF+I: n = 9, N = 3 mice per group).

Appendix Table S1. Quantification of other basic intrinsic properties (resting membrane potential, membrane capacitance, and membrane resistance) of BLA pyramidal neurons recorded from either SPF or GF mice.

	SPF	GF	Significance
**No. of neurons (No. of mice)**	19 (4)	20 (4)	
**Resting membrane potential (mV)**	-68.63 ± 0.97	-69.6 ± 0.97	$P = 0.45$
**Membrane capacitance (pF)**	179.74 ± 6.93	178.25 ± 6.45	$P = 0.49$
**Membrane resistance (MΩ)**	97.26 ± 4.77	105.31 ± 6.17	$P = 0.47$

Appendix Table S2. Detailed information on the statistical analyses (in the order of appearance in the manuscript).

Figure	Number of mice	Age of mice	Statistical test and significance
Fig. 1A	10 SPF, 10 GF	8-14 weeks	Student's *t*-test, $P = 0.0067$
Fig. 1B, 1C	9 SPF, 10 GF	8-14 weeks	Student's *t*-test, (E) $P = 0.0008$; (F) $P = 0.0061$
Fig. 1I	4 SPF, 4 GF	8-14 weeks	Mann-Whitney test, $P = 0.03038$
Fig. 2C	8 cells of 4 SPF, 12 cells of 5 GF	10-14 weeks	Two-way ANOVA, interaction: $F_{(10,180)} = 6.285$, $P < 0.0001$; main effect of current level: $F_{(10, 180)} = 383.4$, $P < 0.0001$; main effect of GF status: $F_{(1, 18)} = 12.48$, $P = 0.0024$
Fig. 2D, 2E	9 cells of 4 SPF, 10 cells of 4 GF	8-14 weeks	Student's T-Test, (D) $P = 0.4226$, (E) $P = 0.5592$
Fig. 2F, 2G	9 cells of 4 SPF, 10 cells of 5 GF	10-14 weeks	Student's *t*-test, (H) $P = 0.0077$, (I) $P = 0.5592$
Fig. 3B	8 cells of 4 SPF, 8 cells of 4 GF	10-14 weeks	Two-way ANOVA, Interaction: $F_{(7, 98)} = 0.6963$, P < 0.6750; Main effect of voltage steps: $F_{(7, 98)} = 117.8$, P < 0.0001; Main effect of GF status: $F_{(1, 14)} = 1.052$, $P = 0.3225$
Fig. 3D	15 cells of 4 SPF, 16 cells of 4 GF	10-14 weeks	Student's *t*-test, $P = 0.0017$
Fig. 3F	10 cells of 4 GF_control, 10 cells of 4 GF_1-EBIO	10-14 weeks	Two-way ANOVA, Interaction: $F_{(10, 180)} = 6.779$, P < 0.0001; Main effect of current steps: $F_{(10, 180)} = 369.3$, $P < 0.0001$; Main effect of GF status: $F_{(1, 18)} = 16.25$, $P = 0.0008$
Fig. 4A	10 GF, 10 CONV	8-14 weeks	Student's *t*-test, $P = 0.0002$
Fig. 4B, 4C	10 GF, 10 CONV	8-14 weeks	Student's *t*-test, (E) $P = 0.0236$; (F) 0.1984
Fig. 4D	12 GF, 12 CONV	8-14 weeks	Student's *t*-test, $P = 0.0135$
Fig. 4E	10 cells of 4 SPF, 12 cells of 4 GF	8-14 weeks	Student's *t*-test, (J) $P = 0.4668$
Fig. 4G	11 cells of 4 SPF, 11 cells of 4 GF	8-14 weeks	Two-way ANOVA, interaction: $F_{(10, 200)} = 5.619$, P < 0.0001; main effect of current step: $F_{(10, 200)} =$

			321.1, P < 0.0001; main effect of GF status: $F_{(1, 20)} = 12.39$, P = 0.0022
Fig. 4I	11 cells of 4 SPF, 12 cells of 4 GF	8-14 weeks	Student's *t*-test, $P = 0.0058$

Fig. 5A<br><br>Fig 5B-D	10 SPF, 9 GF, 8 GF+I<br><br>10 SPF, 8 GF, 8 GF+I	14-16 weeks	Student *t*-test, (A) * $P = 0.0163$; $^{n.s.}P = 0.067$; (B) **$P = 0.0016$;*P = 0.028; (C) ****P <0.0001; **P = 0.0062; (D) **** P <0.0001; **P = 0.0032;
Fig. 5E-F	10 SPF, 6 GF, 7 GF+I	14-16 weeks	Student *t*-test, (E) *$P = 0.0353$; *P = 0.0172; (F) *$P = 0.0163$ (I) **P = 0.002
Fig. 5H	13 cells of 4 SPF, 15 cells of 4 GF	14-15 weeks	Two-way ANOVA, interaction: $F_{(10, 200)} = 5.74$, $P < 0.0001$; main effect of current step: $F_{(10, 200)}$ = 200.36, $P < 0.0001$; main effect of GF status: $F_{(1, 20)} = 194.88$, P < 0.0001
Fig. 5I	15 cells of 4 SPF, 13 cells of 4 GF	14-15 weeks	Student's *t*-test, $P = 0.0387$
Fig. 5J	13 cells of 4 SPF, 15 cells of 4 GF	14-16 weeks	Two-way ANOVA, interaction: $F_{(10, 200)} = 3.76$, $P < 0.0001$; main effect of current step: $F_{(10, 200)}$ = 196.78, $P < 0.0001$; main effect of GF status: $F_{(1, 20)} = 100.66$, P < 0.0001
Fig. S2A	12 cells of 4 SPF, 11 cells of 4 GF	8-14 weeks	Two way ANOVA, Interaction: $F_{(14, 308)} = 0.1275$, $P > 0.9999$; the Main effect of voltage steps: $F_{(14, 308)}$ = 83.89, $P < 0.0001$; Main effect of GF status: $F_{(1, 22)} = 0.0211$, $P = 0.8858$
Fig. S2B	12 cells of 4 SPF, 11 cells of 4 GF	8-14 weeks	Two way ANOVA, Interaction: $F_{(14, 308)} = 0.6963$, $P = 0.456$; Main effect of voltage steps: $F_{(14, 308)}$ = 220.5, $P < 0.0001$; Main effect of GF status: $F_{(1, 22)} = 1.419$, P = 0.2463
Fig. S2H	9 cells of 4 SPF_control,<br>10 cells of 4 SPF_apamin	8-14 weeks	Student's T-Test, $P = 0.5592$

Fig. S3D	9 cells of 4 SPF, 9 cells of 4 GF	8-14 weeks	Two-way ANOVA, Interaction: $F_{(10, 160)} = 4.036$, $P < 0.0001$; Main effect of current steps: $F_{(10, 160)} = 401.7$, $P < 0.0001$; Main effect of GF status: $F_{(1, 16)} = 10.33$, $P = 0.0054$
Fig. S3F	8 cells of 3 SPF, 8 cells of 3 GF	8-14 weeks	Two-way ANOVA, interaction: $F_{(10, 140)} = 0.8881$, $P < 0.546$; main effect of current steps: $F_{(10, 140)} =$
			432.2, $P < 0.0001$; main effect of GF status: $F_{(1, 14)} = 19.93$, $P = 0.3154$
Fig. S5G	4 SPF, 4 GF	8-14 weeks	Mann-Whitney test, $P = 0.03$
