# Supplementary figures and images for: Microbial metabolites tune amygdala neuronal hyperexcitability and anxiety-linked behaviors
Source: EMBO Mol Med. 2025 Feb 5;17(2):249–64. doi: 10.1038/s44321-024-00179-y (PMC11821874; doi:10.1038/s44321-024-00179-y)

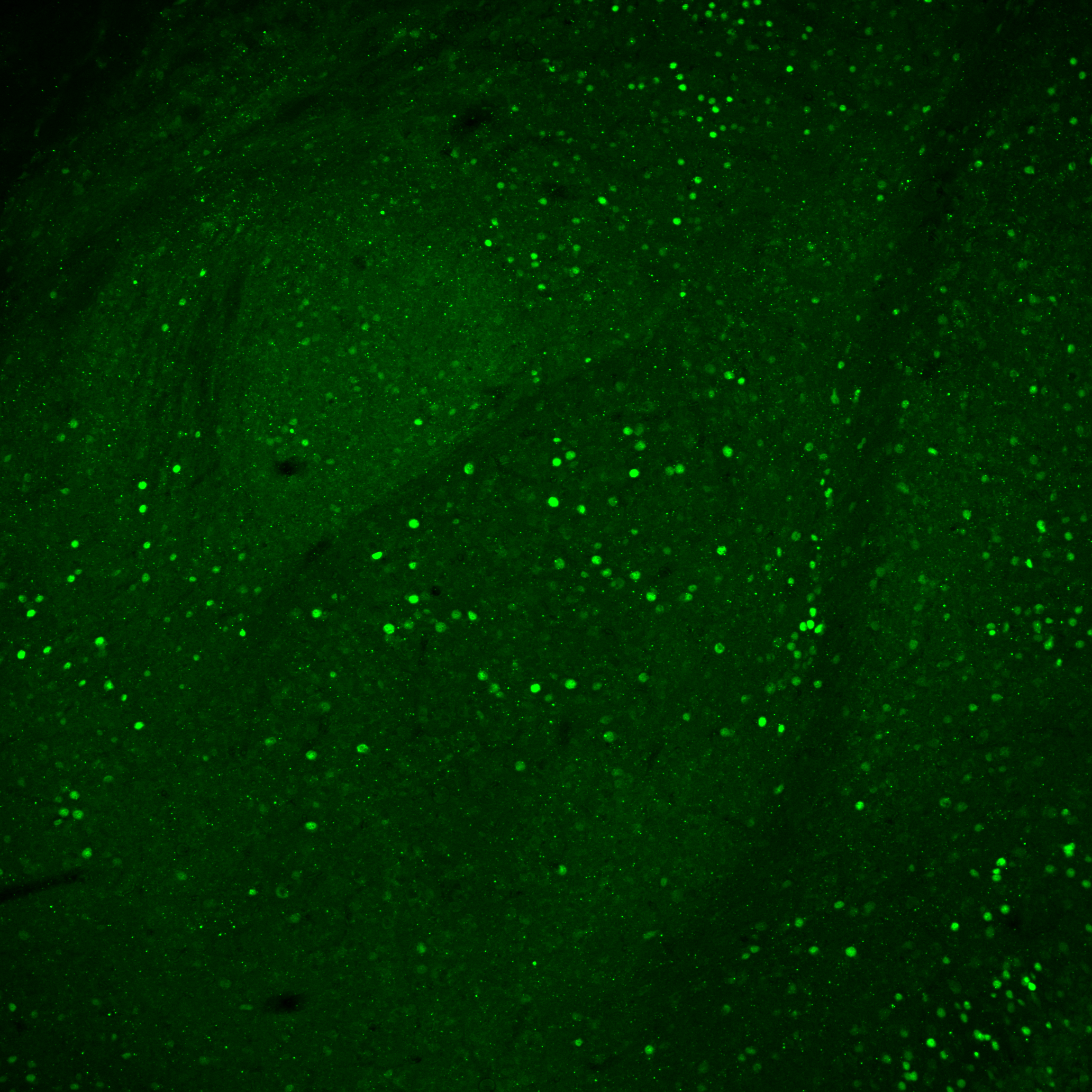

Supplement: Supplementary file 3 — Source data Fig. 1 [file 44321_2024_179_MOESM3_ESM.zip › 1E(Amygdala)/GF/10_cFos.png]

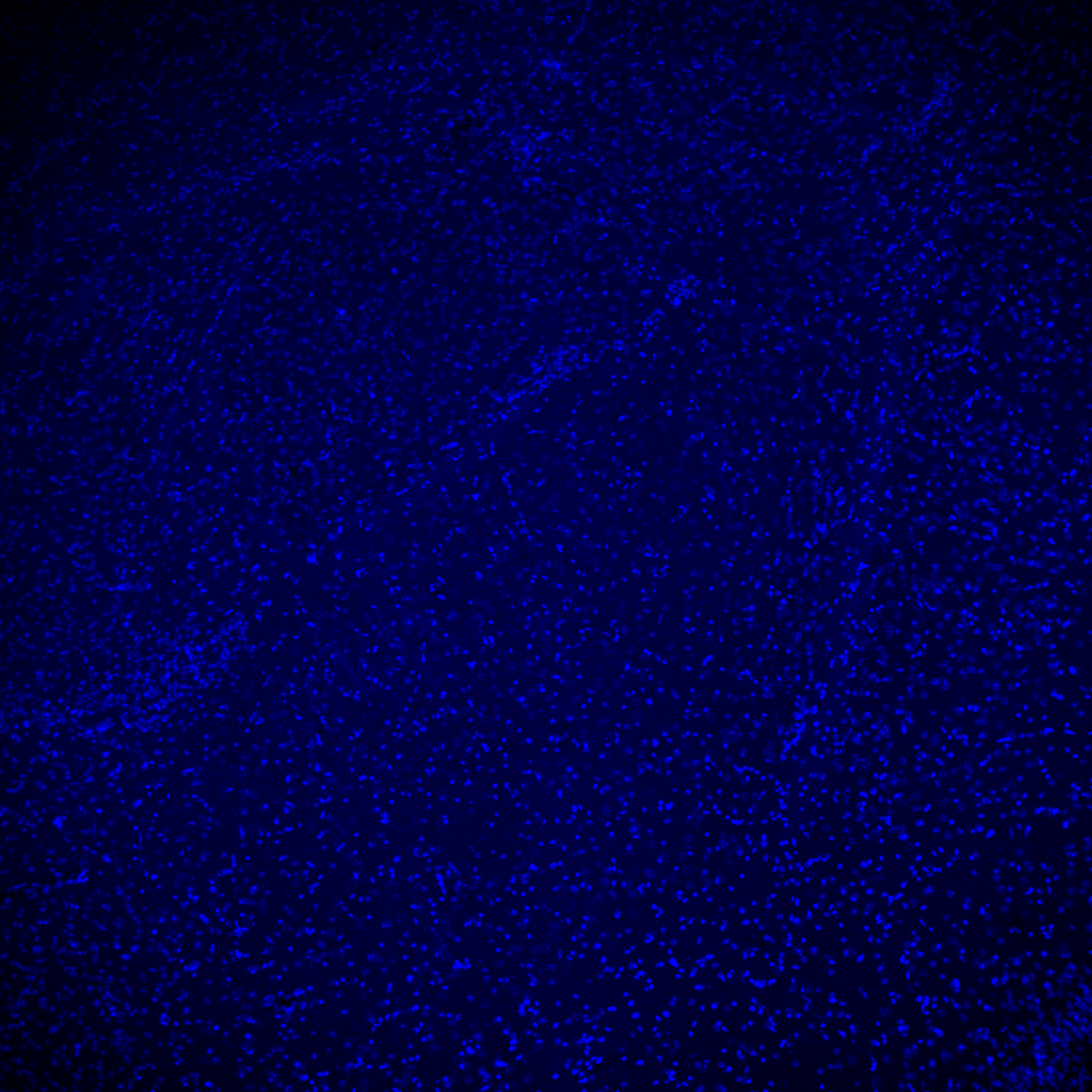

Supplement: Supplementary file 3 — Source data Fig. 1 [file 44321_2024_179_MOESM3_ESM.zip › 1E(Amygdala)/GF/10_DAPI.png]

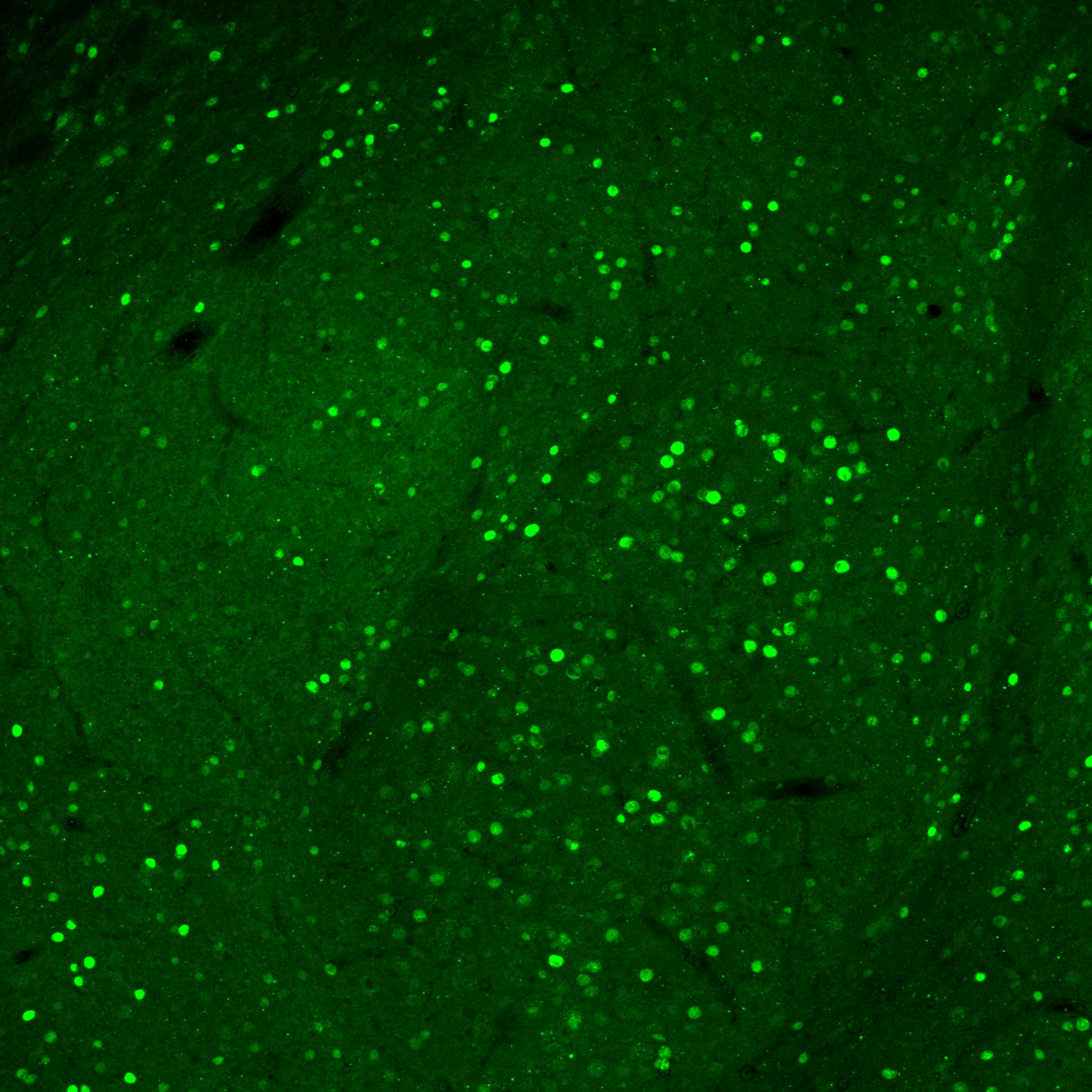

Supplement: Supplementary file 3 — Source data Fig. 1 [file 44321_2024_179_MOESM3_ESM.zip › 1E(Amygdala)/GF/1_cFos.png]

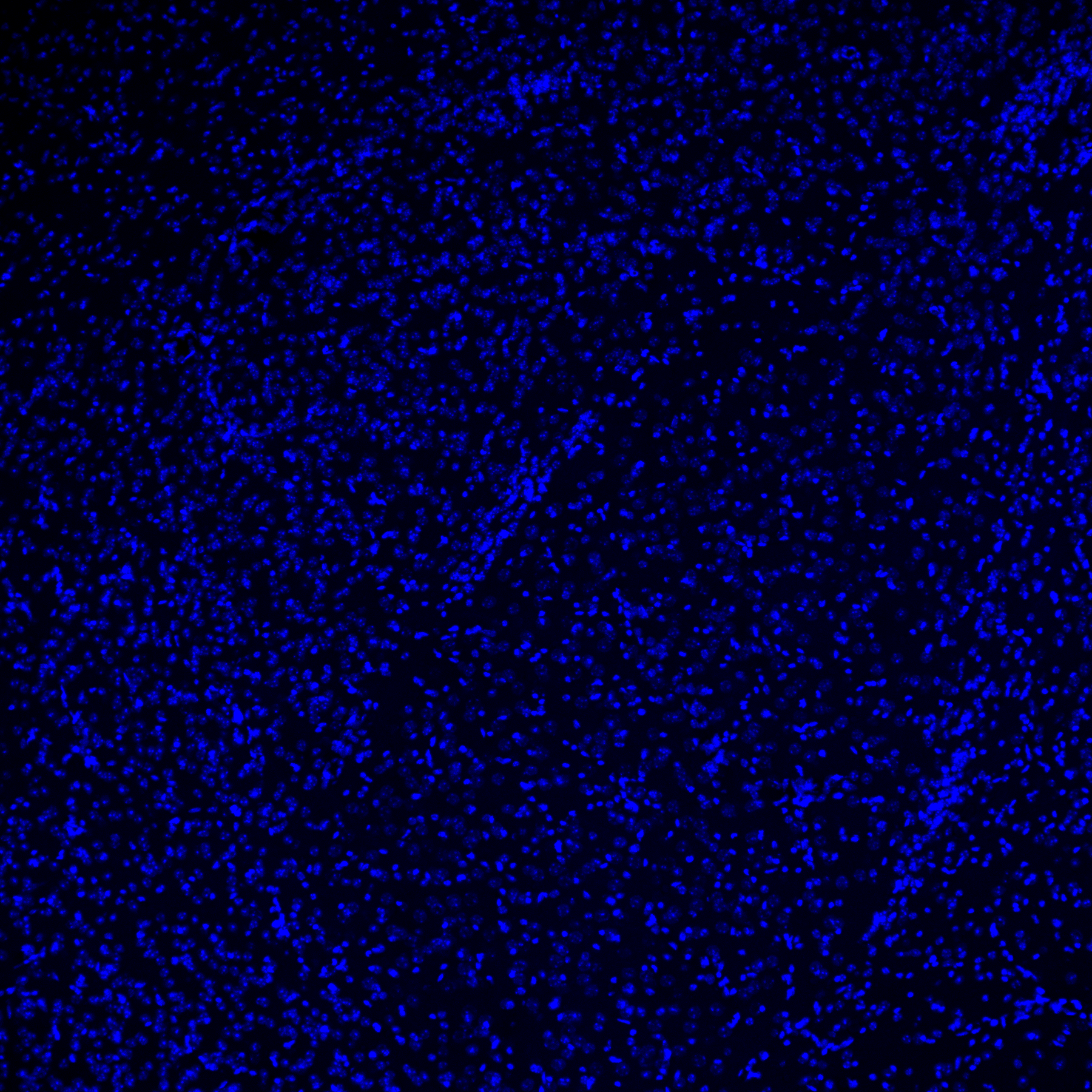

Supplement: Supplementary file 3 — Source data Fig. 1 [file 44321_2024_179_MOESM3_ESM.zip › 1E(Amygdala)/GF/1_DAPI.png]

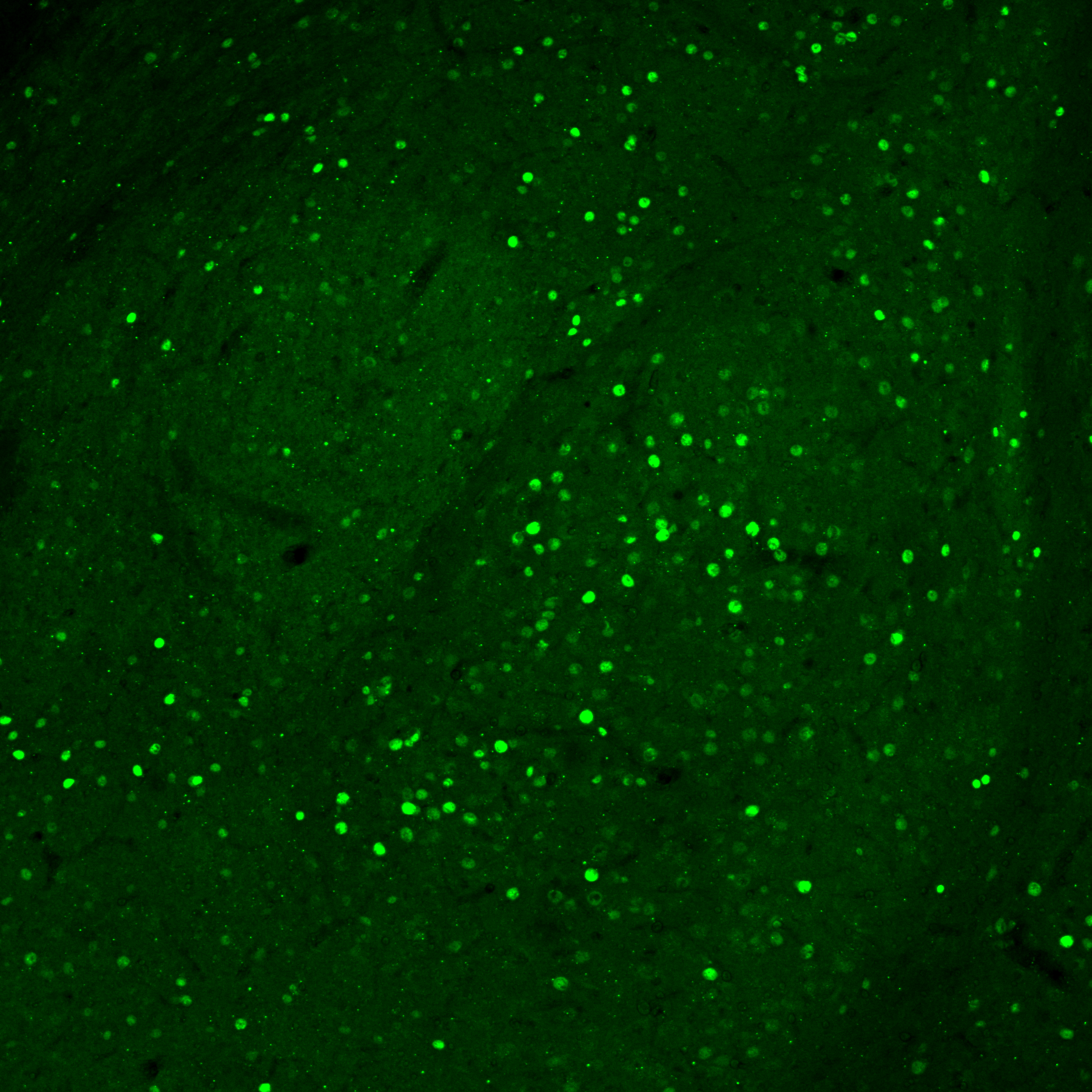

Supplement: Supplementary file 3 — Source data Fig. 1 [file 44321_2024_179_MOESM3_ESM.zip › 1E(Amygdala)/GF/2_cFos.png]

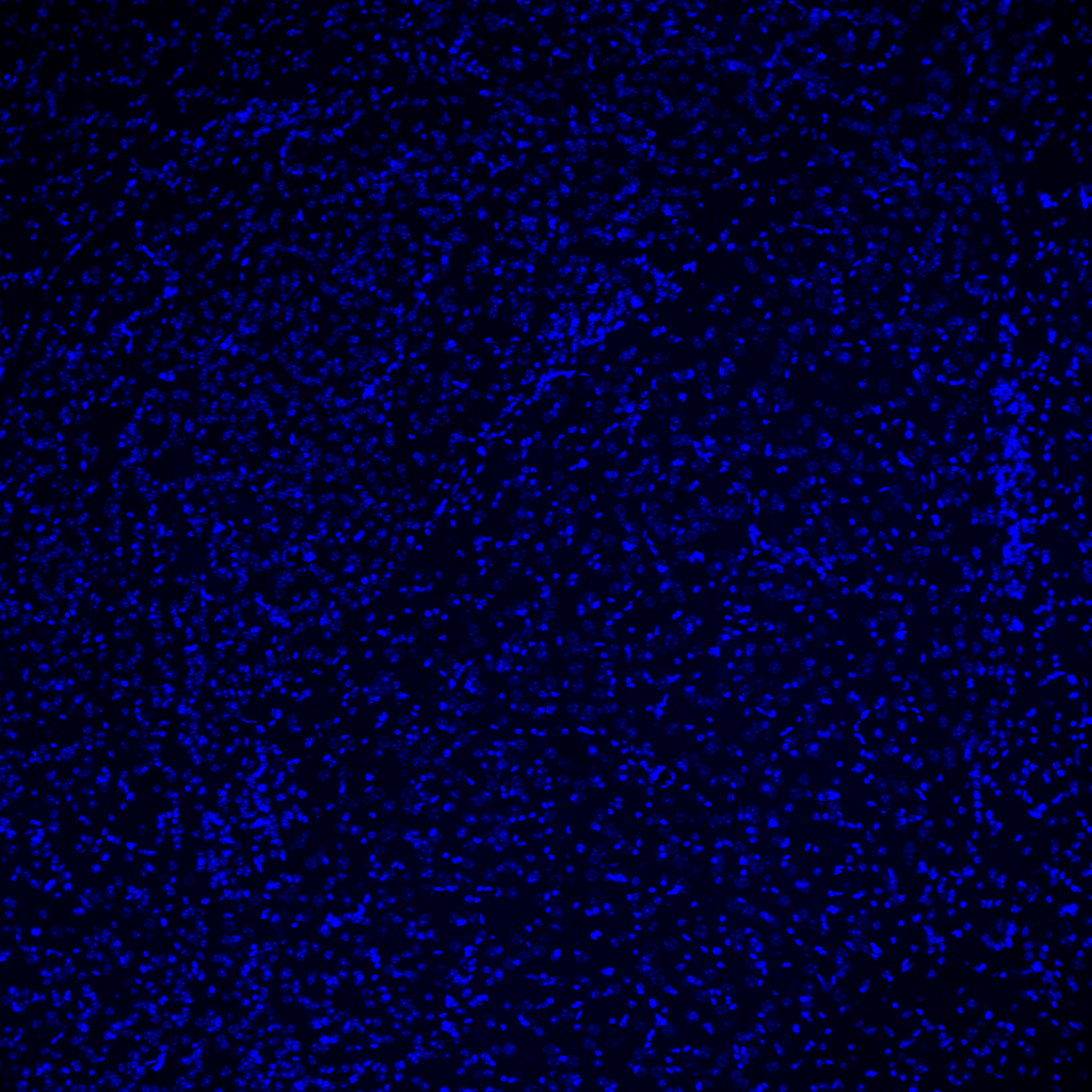

Supplement: Supplementary file 3 — Source data Fig. 1 [file 44321_2024_179_MOESM3_ESM.zip › 1E(Amygdala)/GF/2_DAPI.png]

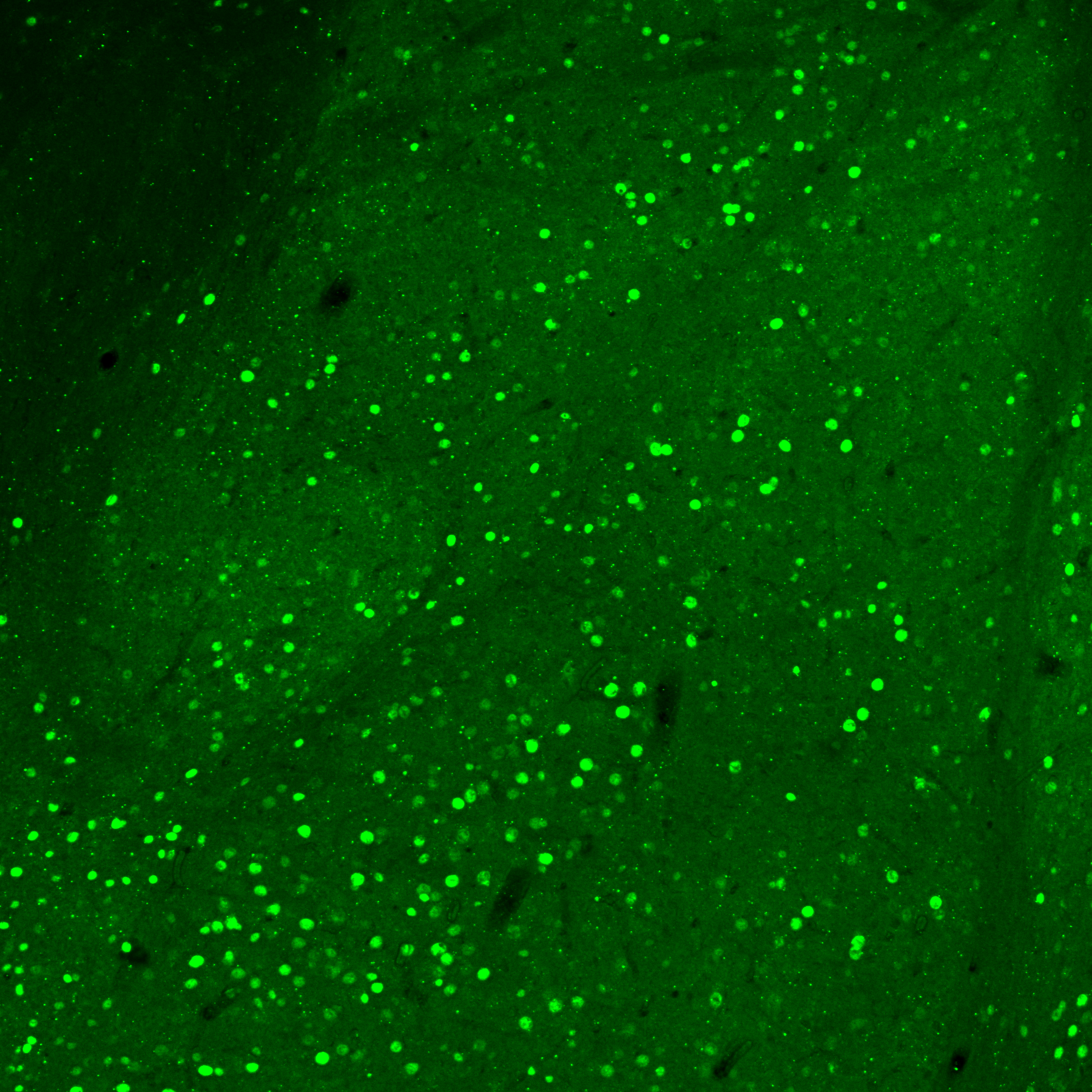

Supplement: Supplementary file 3 — Source data Fig. 1 [file 44321_2024_179_MOESM3_ESM.zip › 1E(Amygdala)/GF/5_cFos.png]

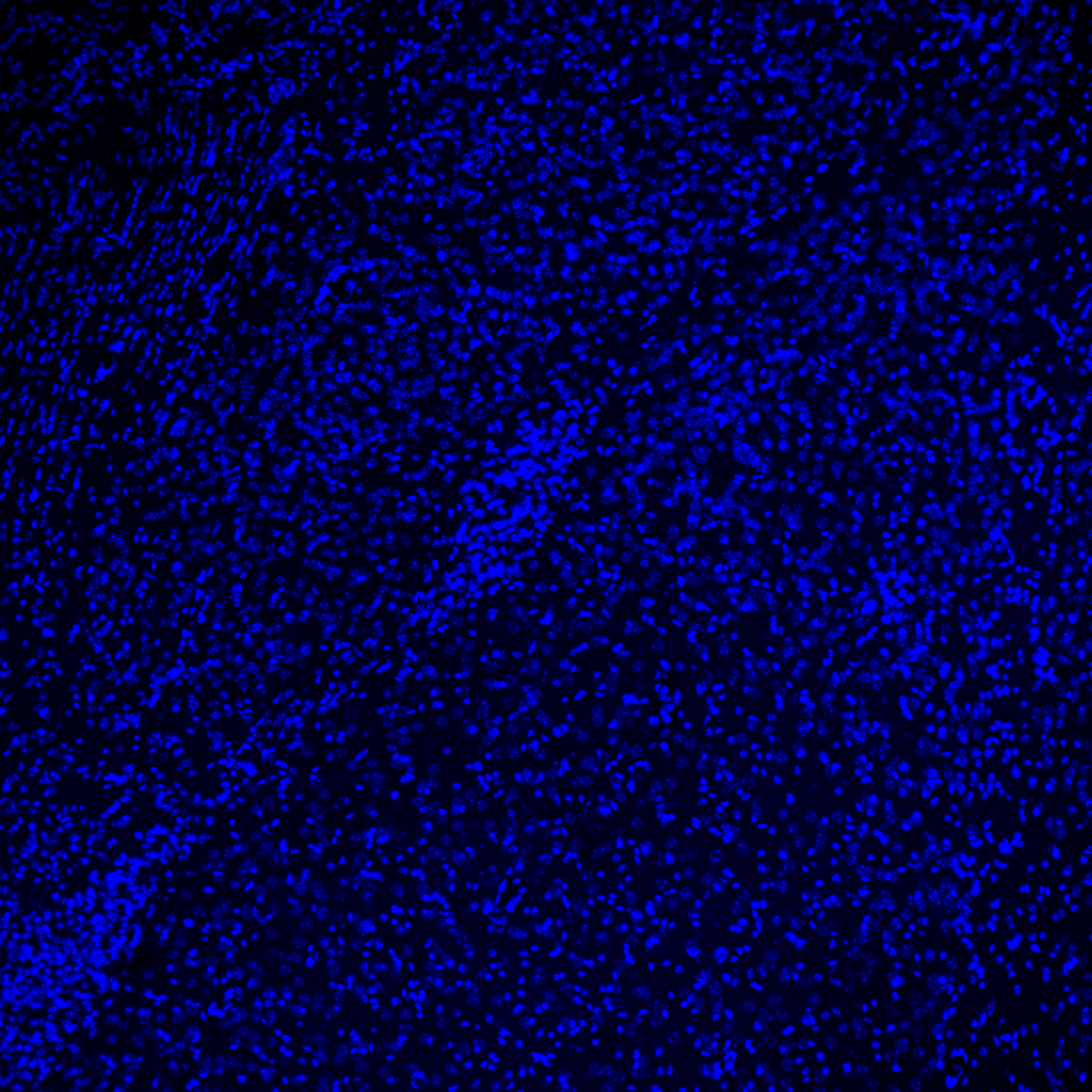

Supplement: Supplementary file 3 — Source data Fig. 1 [file 44321_2024_179_MOESM3_ESM.zip › 1E(Amygdala)/GF/5_DAPI.png]

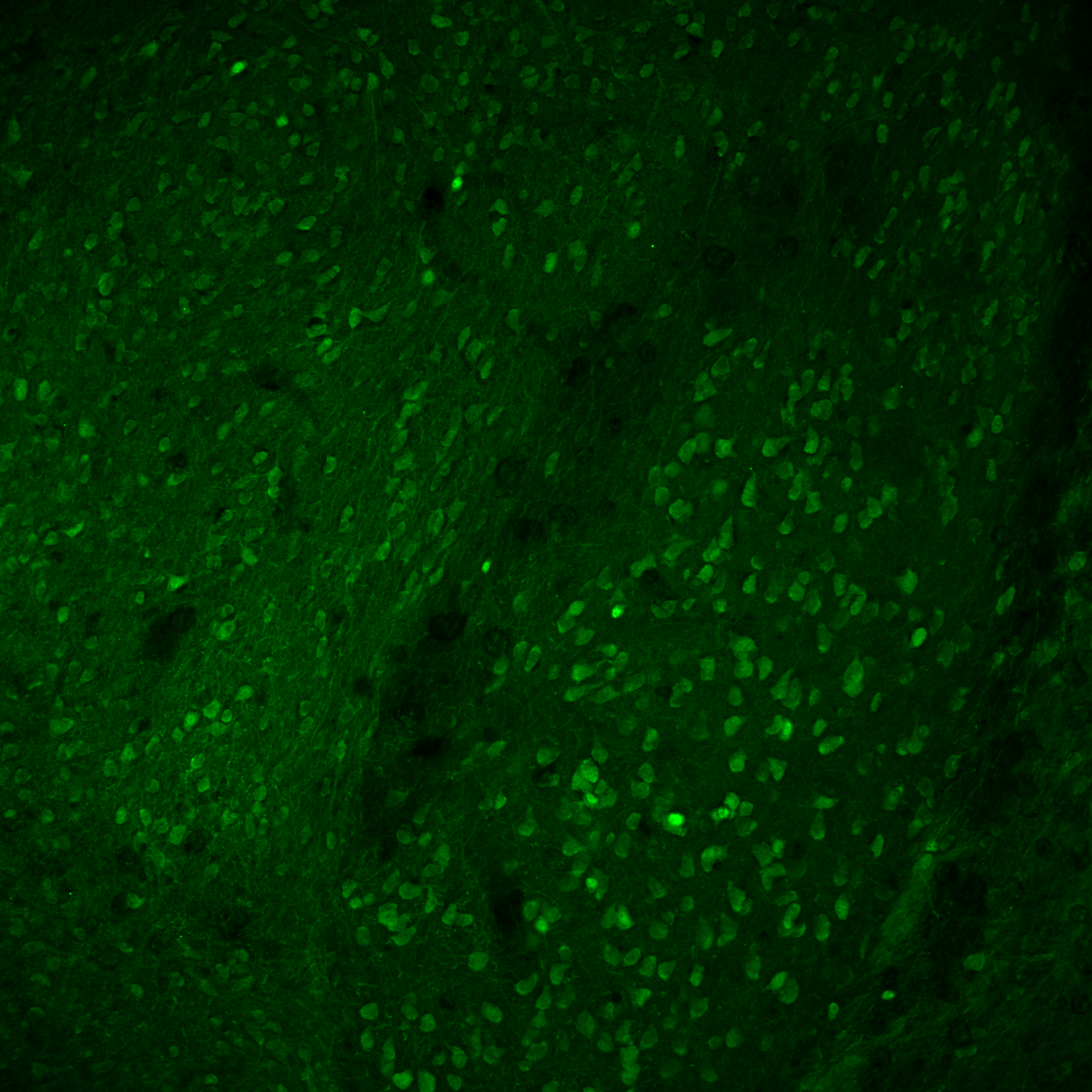

Supplement: Supplementary file 3 — Source data Fig. 1 [file 44321_2024_179_MOESM3_ESM.zip › 1E(Amygdala)/SPF/1_cFos.png]

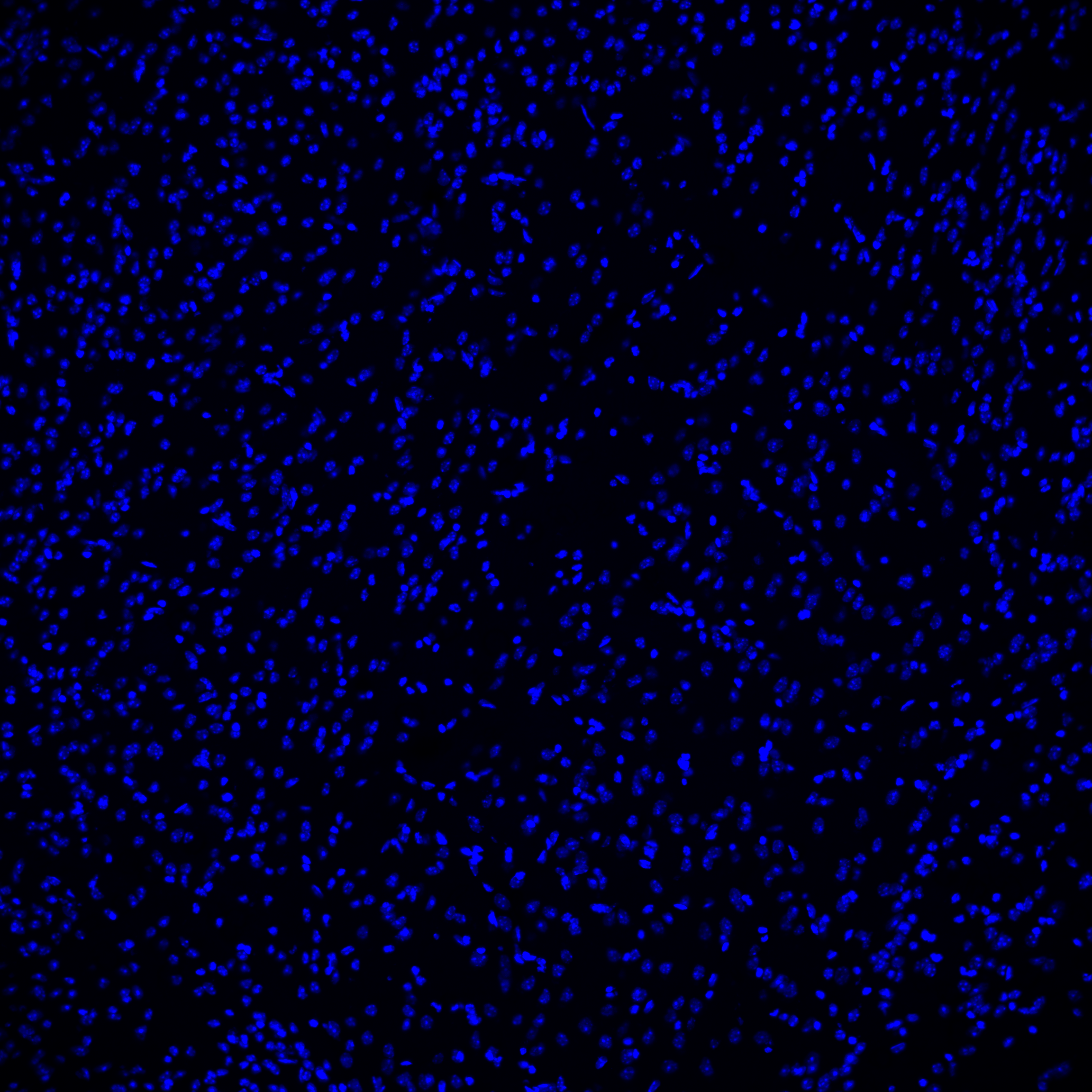

Supplement: Supplementary file 3 — Source data Fig. 1 [file 44321_2024_179_MOESM3_ESM.zip › 1E(Amygdala)/SPF/1_DAPI.png]

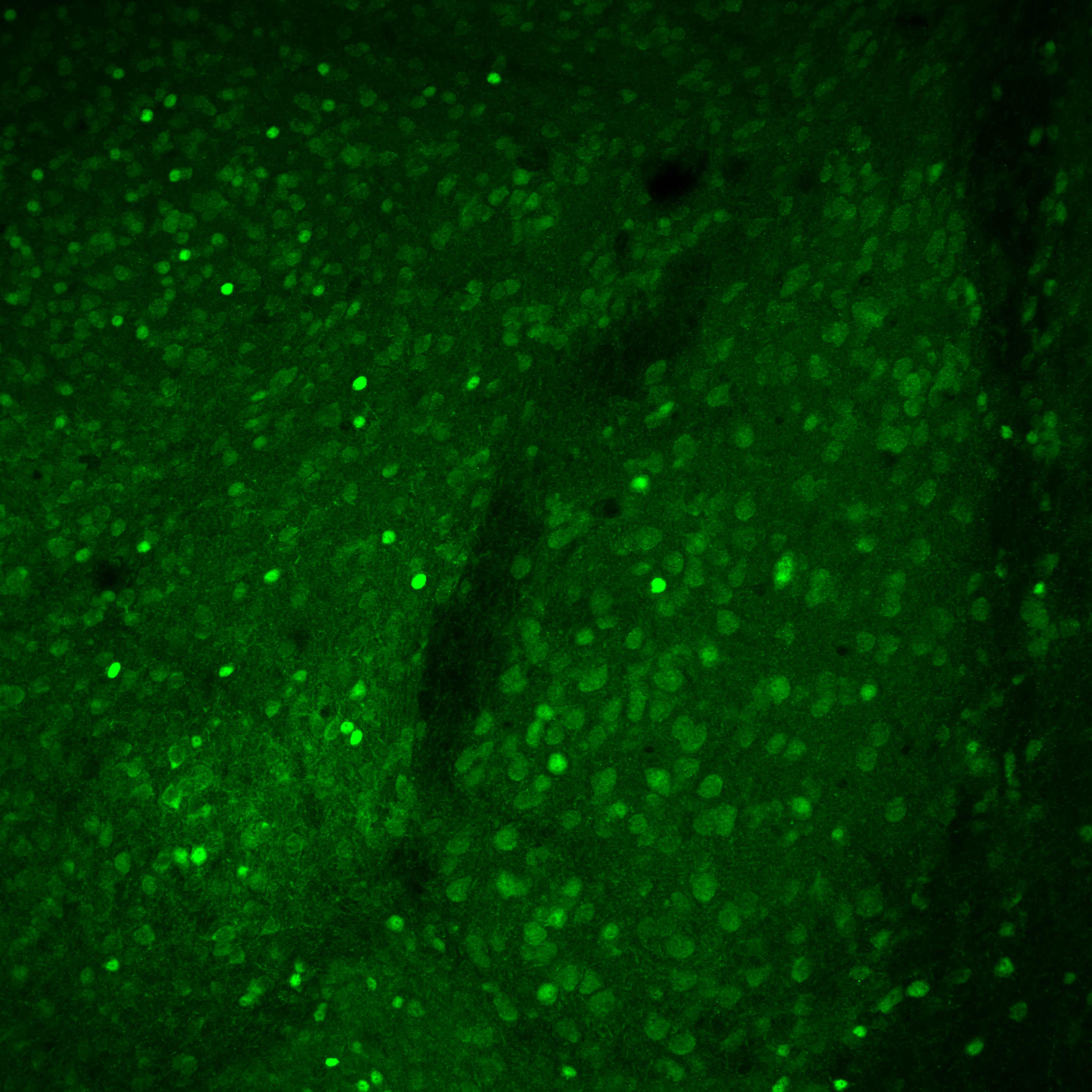

Supplement: Supplementary file 3 — Source data Fig. 1 [file 44321_2024_179_MOESM3_ESM.zip › 1E(Amygdala)/SPF/6_cFos.png]

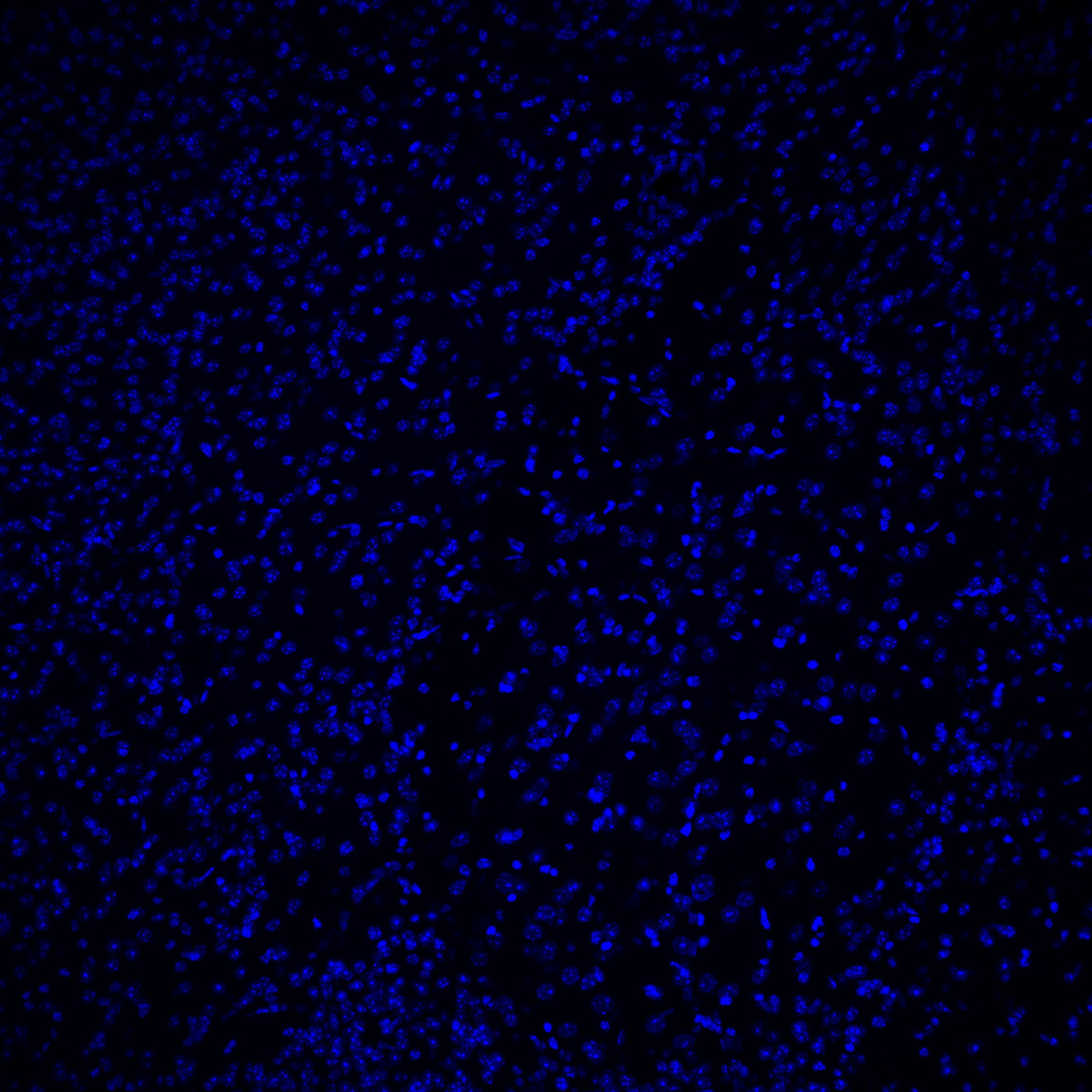

Supplement: Supplementary file 3 — Source data Fig. 1 [file 44321_2024_179_MOESM3_ESM.zip › 1E(Amygdala)/SPF/6_DAPI.png]

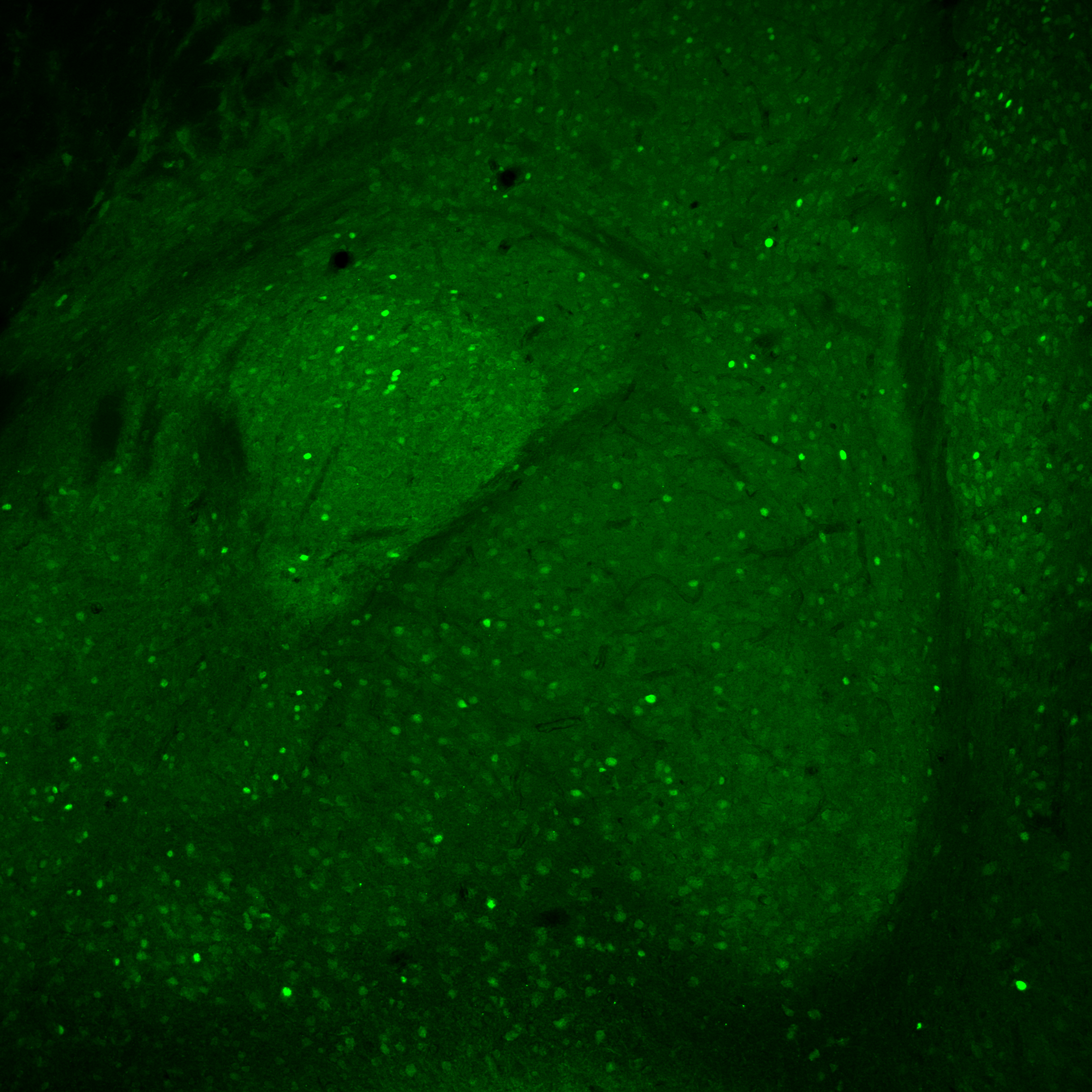

Supplement: Supplementary file 3 — Source data Fig. 1 [file 44321_2024_179_MOESM3_ESM.zip › 1E(Amygdala)/SPF/8_cFos.png]

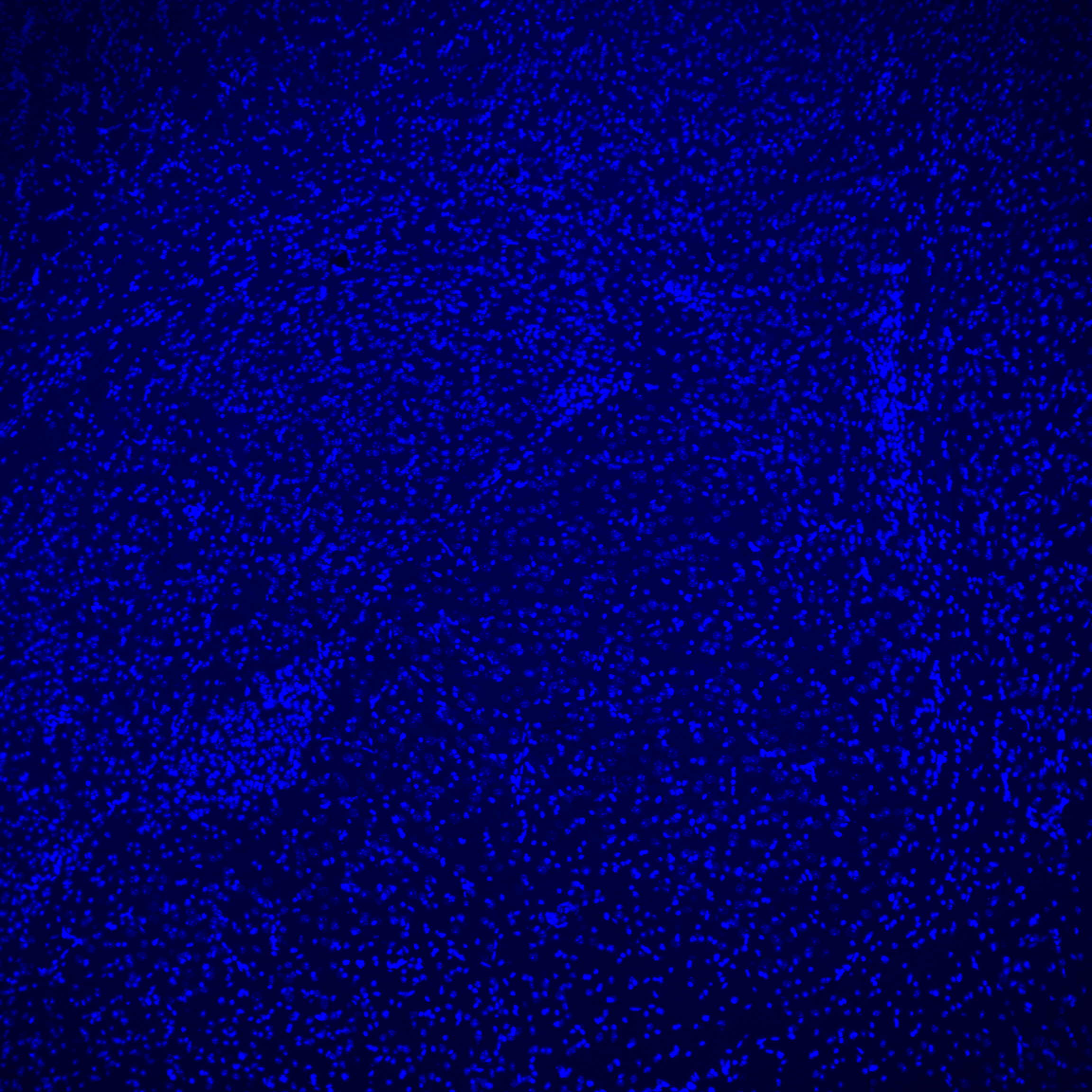

Supplement: Supplementary file 3 — Source data Fig. 1 [file 44321_2024_179_MOESM3_ESM.zip › 1E(Amygdala)/SPF/8_DAPI.png]

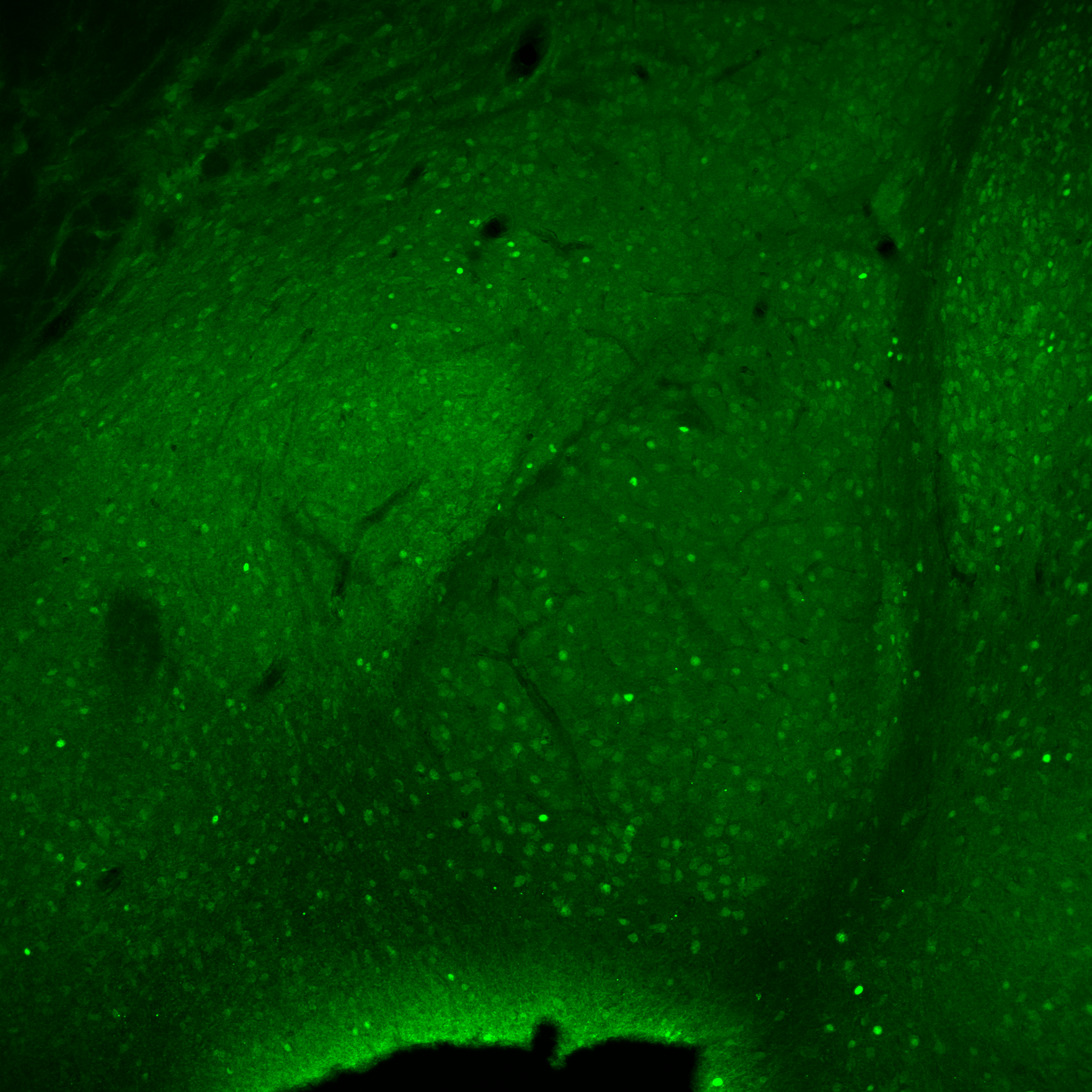

Supplement: Supplementary file 3 — Source data Fig. 1 [file 44321_2024_179_MOESM3_ESM.zip › 1E(Amygdala)/SPF/9_cFos.png]

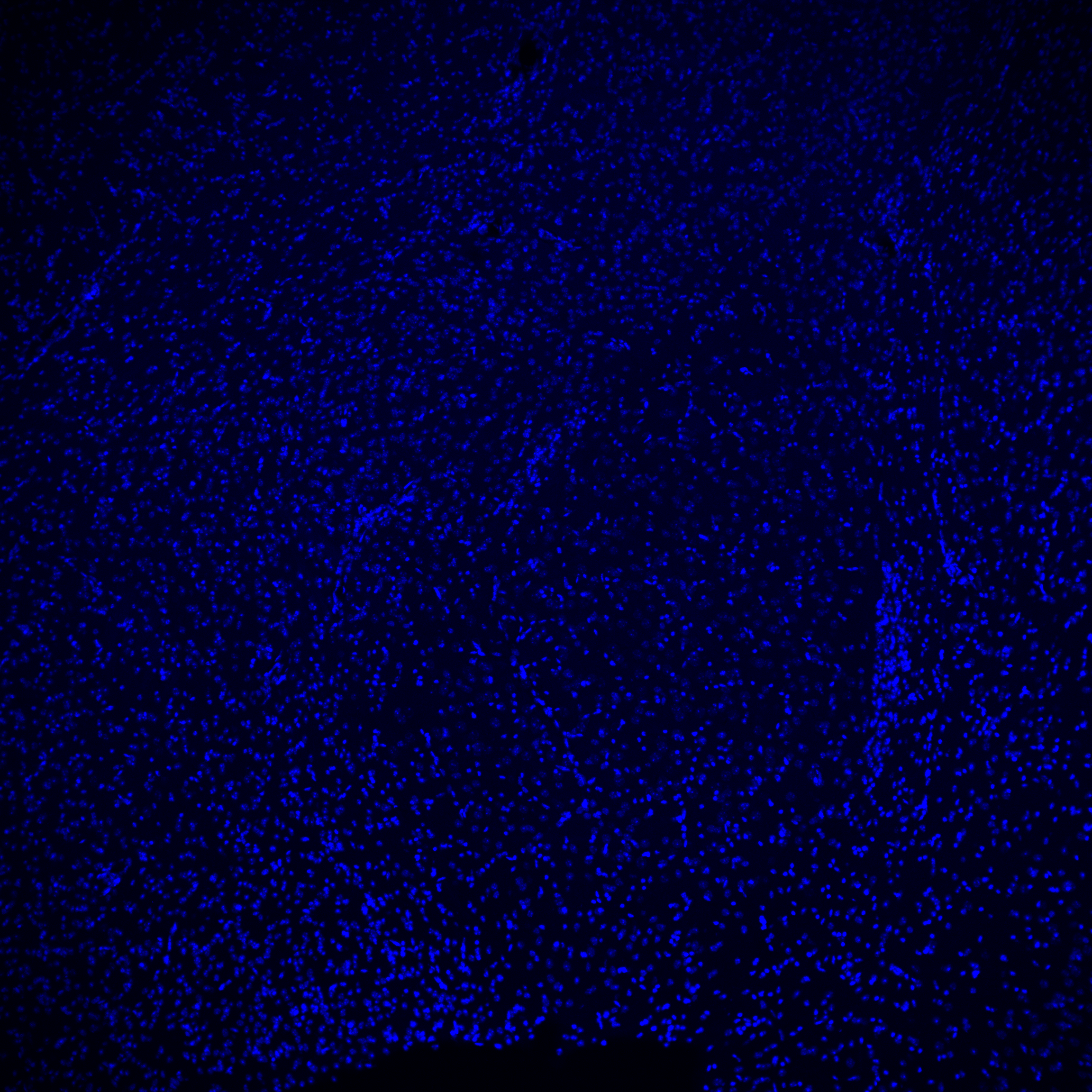

Supplement: Supplementary file 3 — Source data Fig. 1 [file 44321_2024_179_MOESM3_ESM.zip › 1E(Amygdala)/SPF/9_DAPI.png]

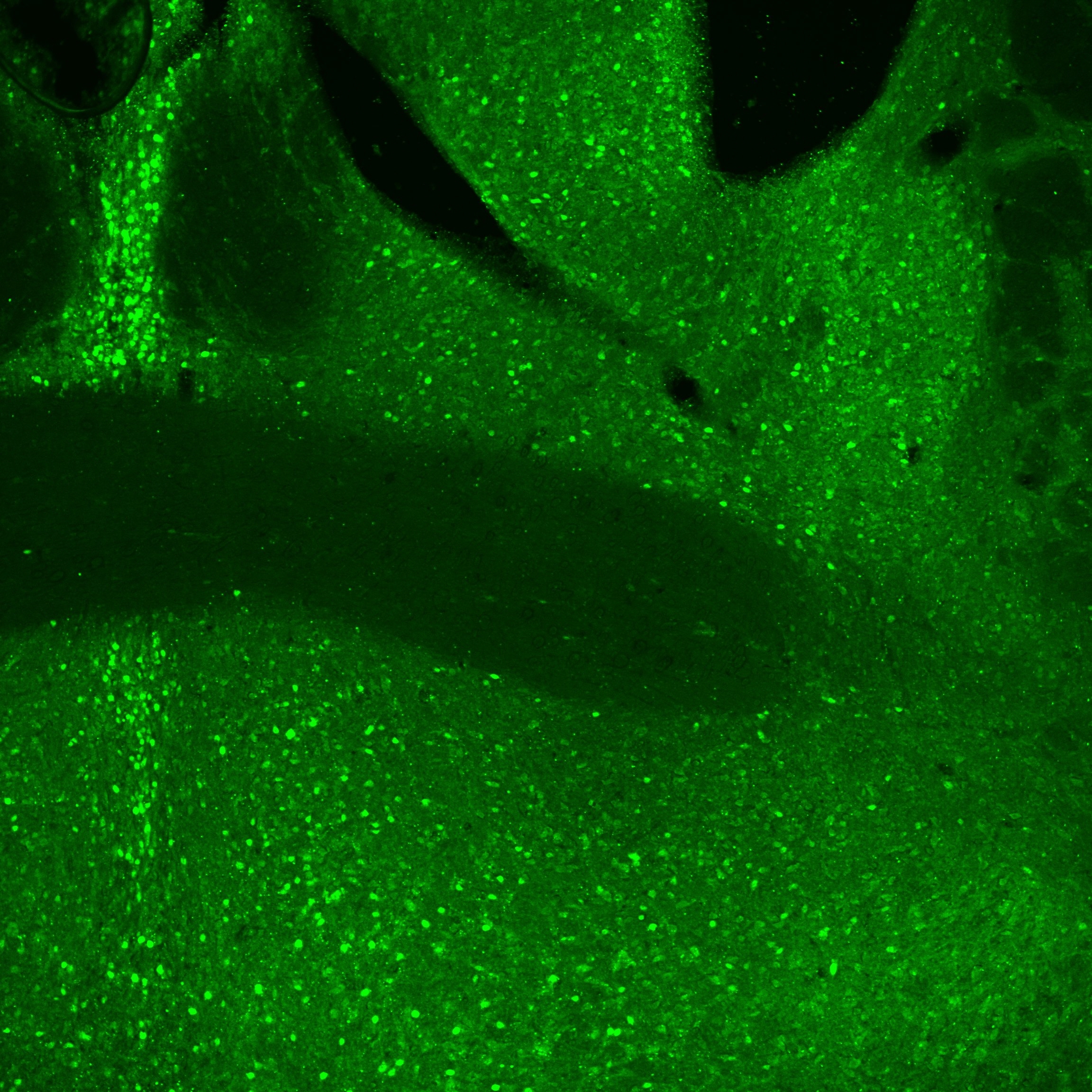

Supplement: Supplementary file 3 — Source data Fig. 1 [file 44321_2024_179_MOESM3_ESM.zip › 1F-20241022T063939Z-001/1F/GF/1F_BNST.jpg]

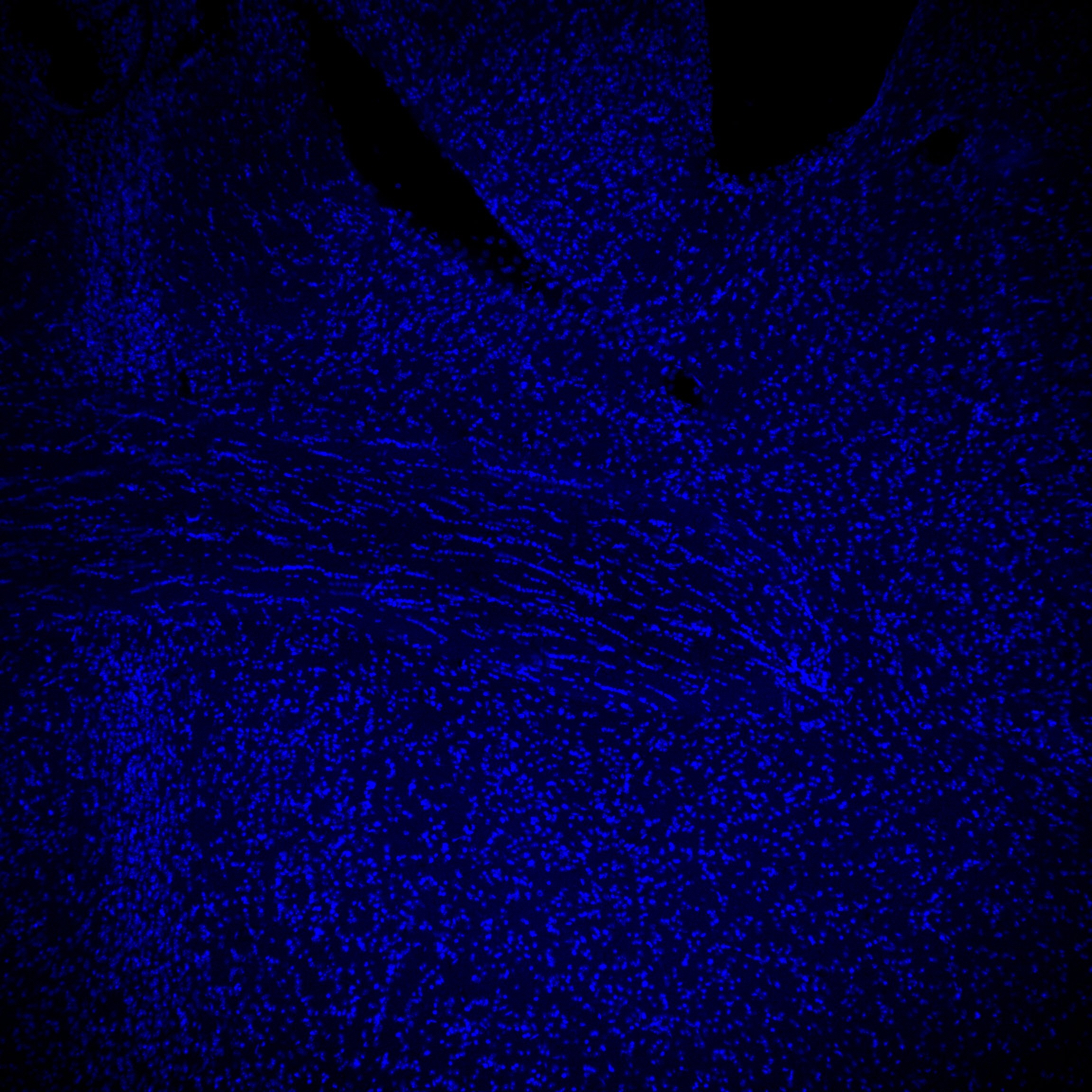

Supplement: Supplementary file 3 — Source data Fig. 1 [file 44321_2024_179_MOESM3_ESM.zip › 1F-20241022T063939Z-001/1F/GF/1F_BNST_DAPI.jpg]

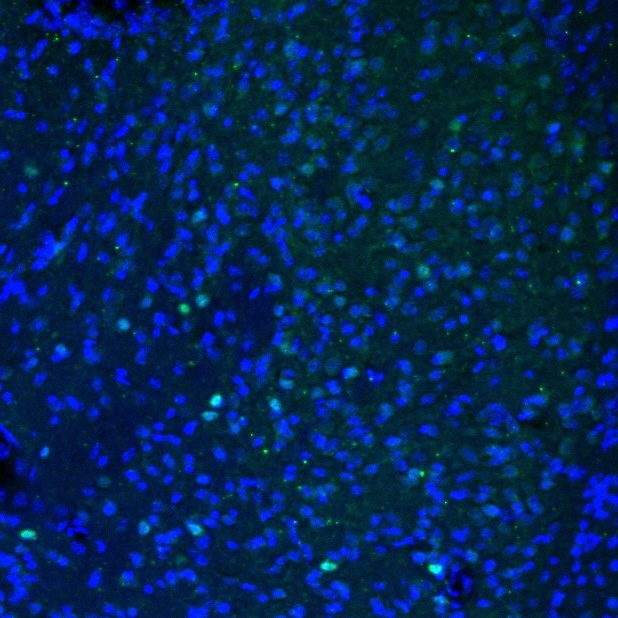

Supplement: Supplementary file 3 — Source data Fig. 1 [file 44321_2024_179_MOESM3_ESM.zip › 1F-20241022T063939Z-001/1F/GF/1F_cropped.jpg]

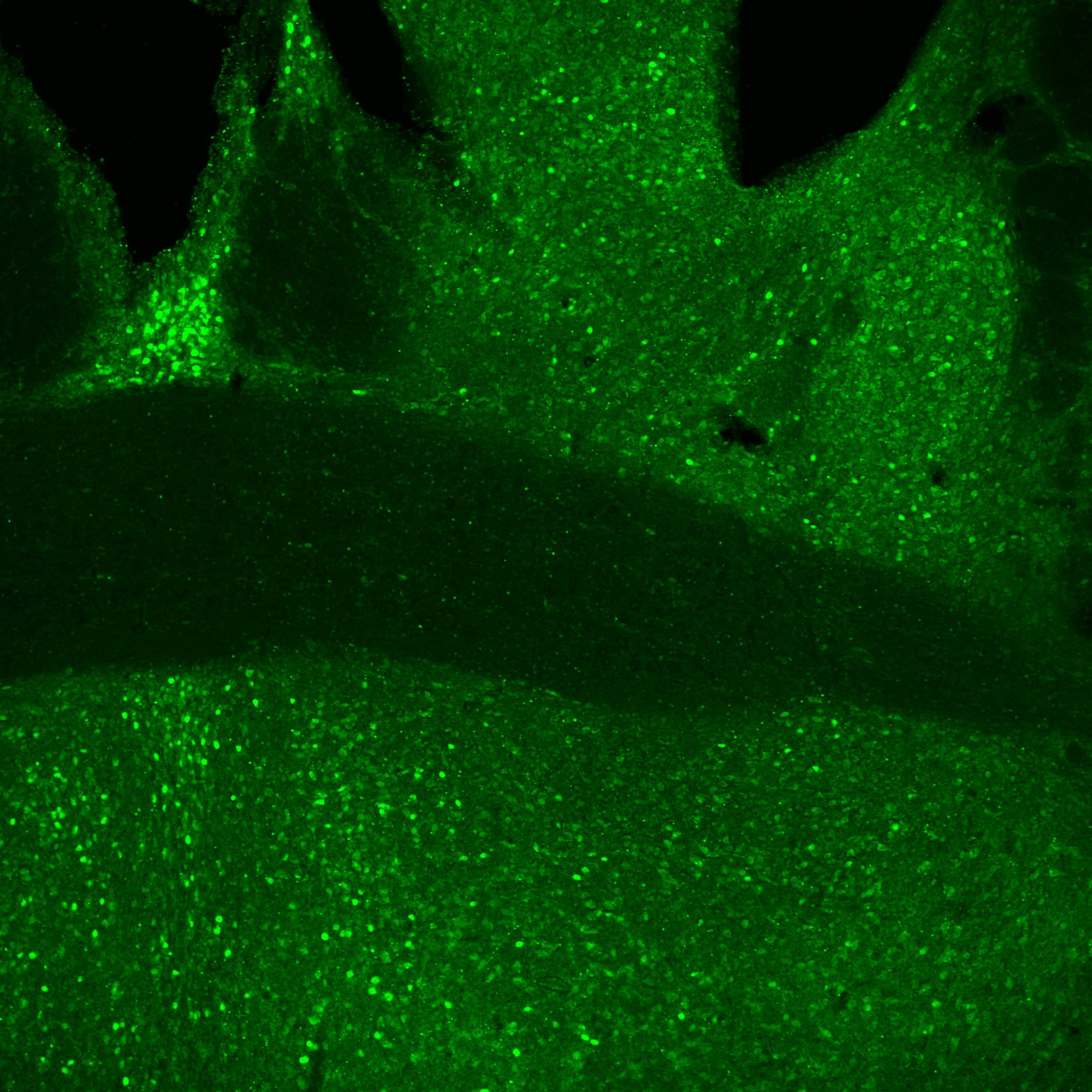

Supplement: Supplementary file 3 — Source data Fig. 1 [file 44321_2024_179_MOESM3_ESM.zip › 1F-20241022T063939Z-001/1F/SPF/1F_BNST.jpg]

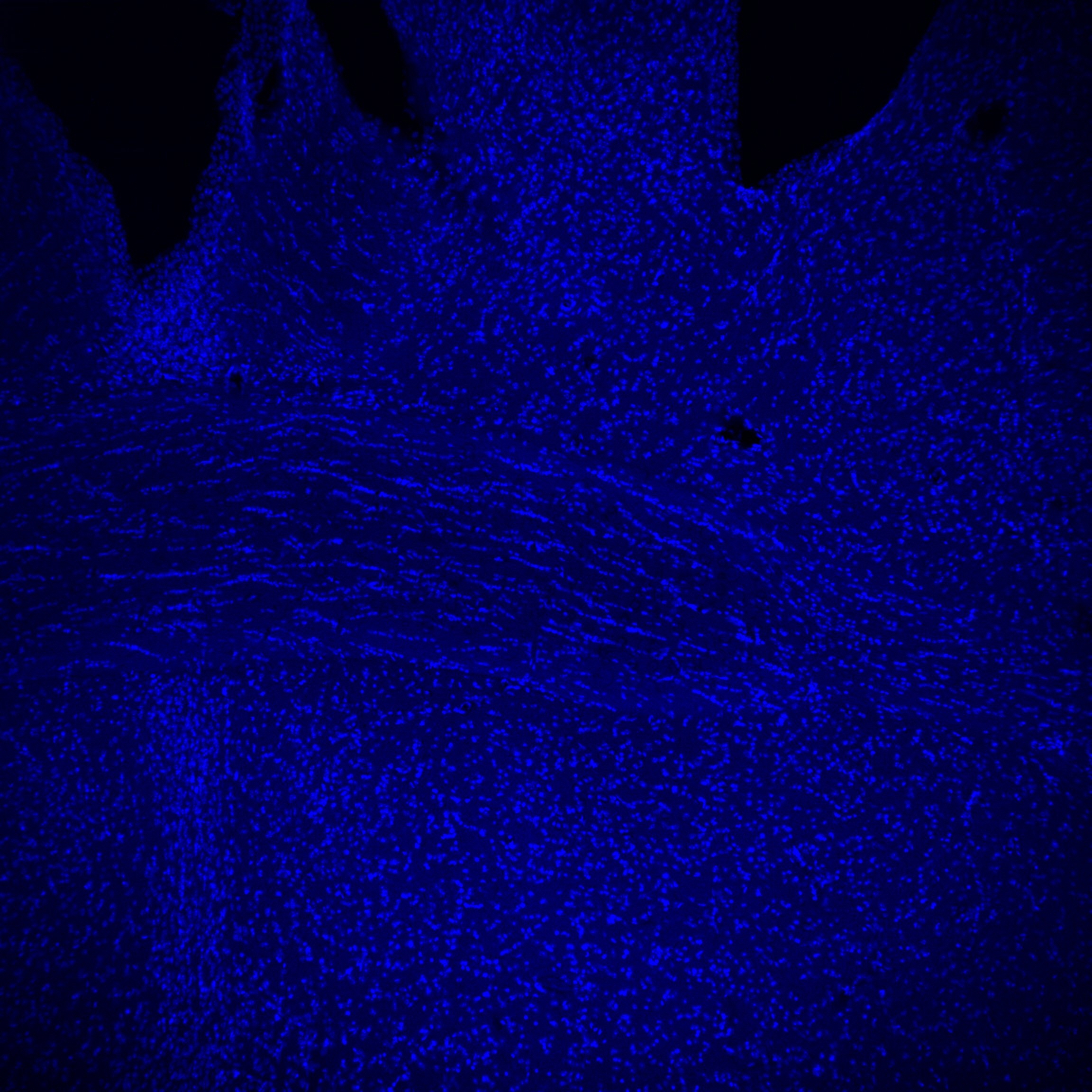

Supplement: Supplementary file 3 — Source data Fig. 1 [file 44321_2024_179_MOESM3_ESM.zip › 1F-20241022T063939Z-001/1F/SPF/1F_BNST_DAPI.jpg]

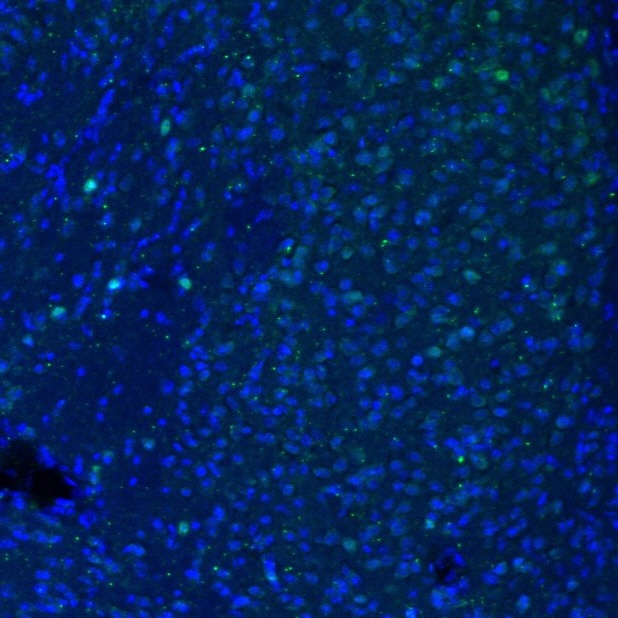

Supplement: Supplementary file 3 — Source data Fig. 1 [file 44321_2024_179_MOESM3_ESM.zip › 1F-20241022T063939Z-001/1F/SPF/1F_cropped.jpg]

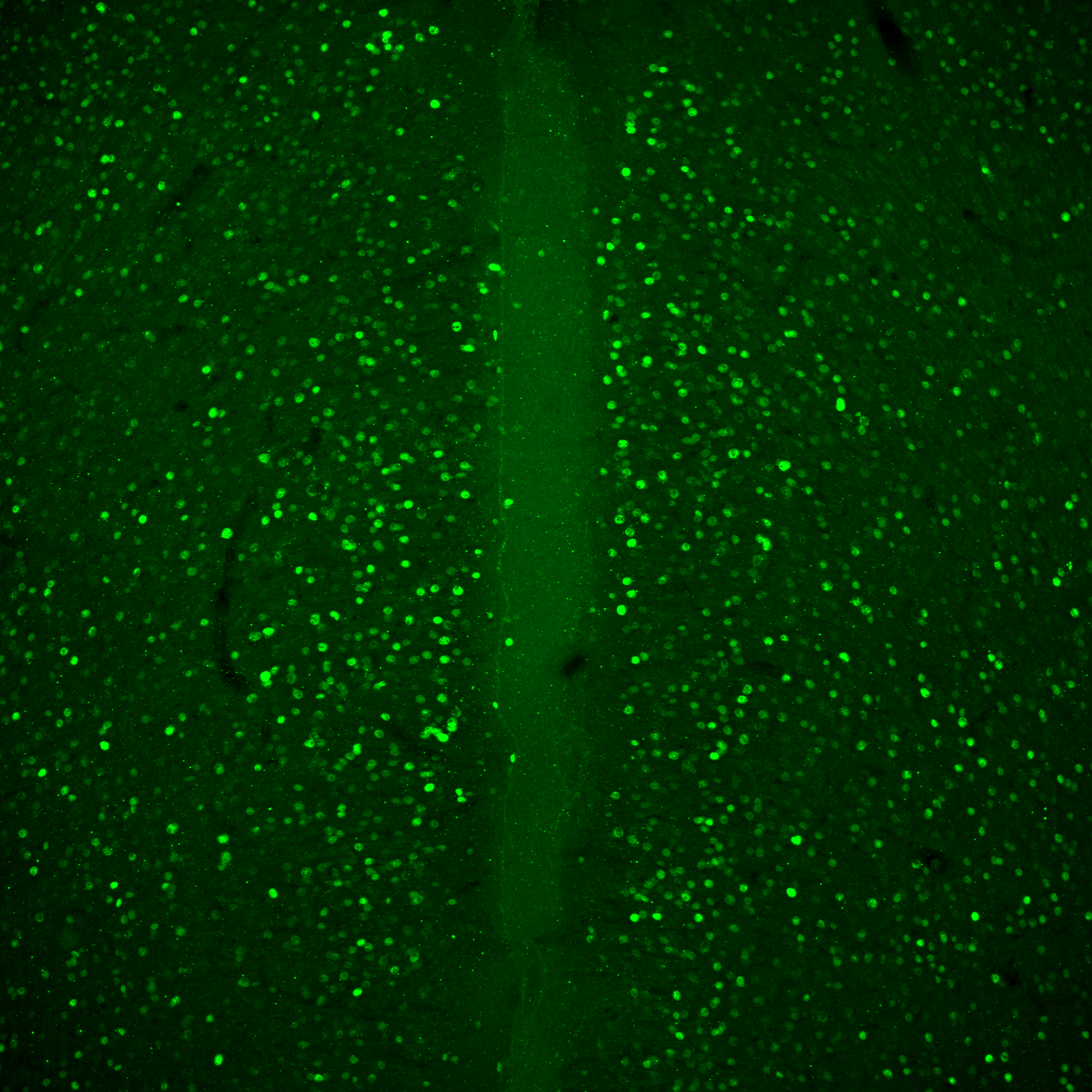

Supplement: Supplementary file 3 — Source data Fig. 1 [file 44321_2024_179_MOESM3_ESM.zip › 1G-20241022T063913Z-001/1G/GF/1_cFos.png]

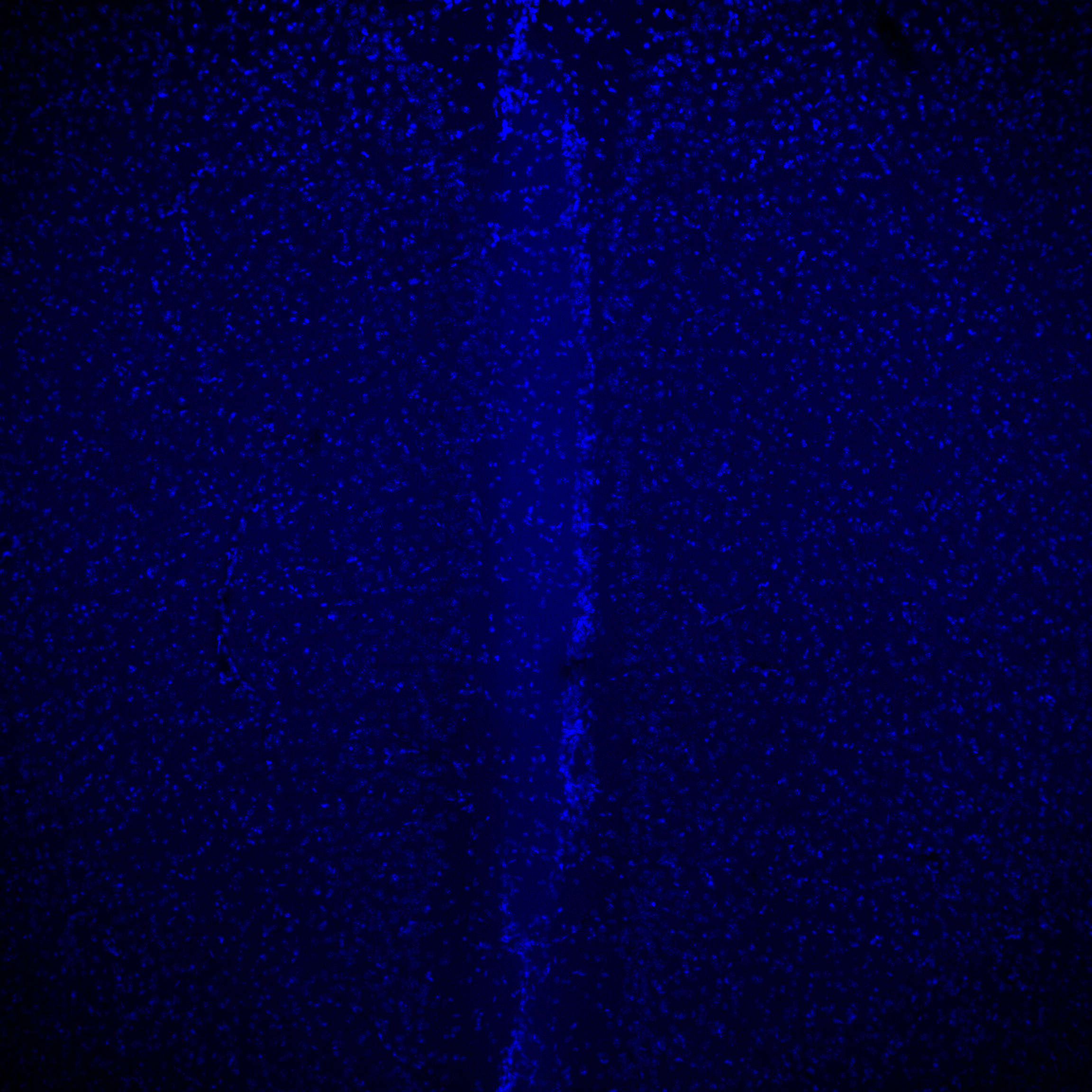

Supplement: Supplementary file 3 — Source data Fig. 1 [file 44321_2024_179_MOESM3_ESM.zip › 1G-20241022T063913Z-001/1G/GF/1_DAPI.png]

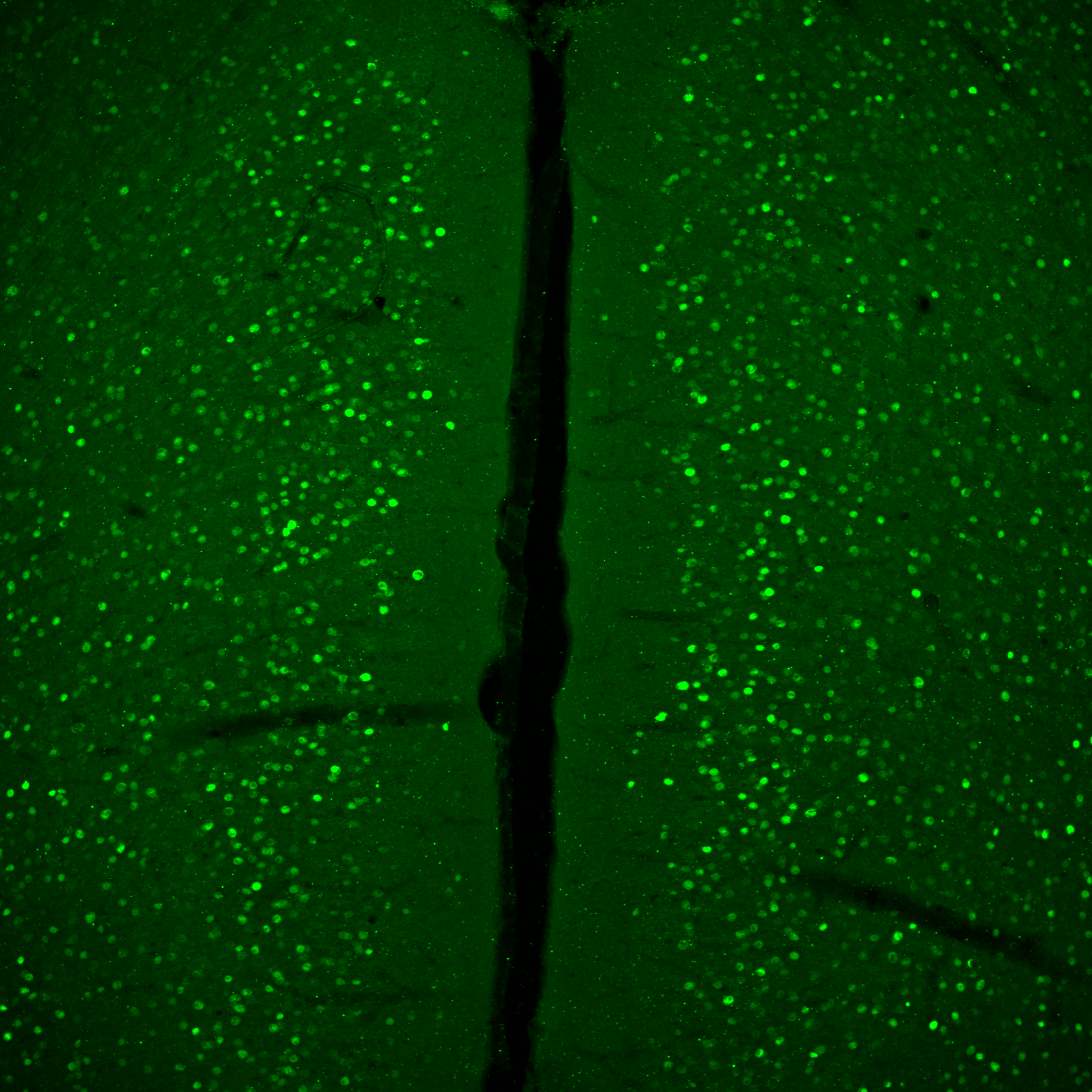

Supplement: Supplementary file 3 — Source data Fig. 1 [file 44321_2024_179_MOESM3_ESM.zip › 1G-20241022T063913Z-001/1G/SPF/1_cFos.png]

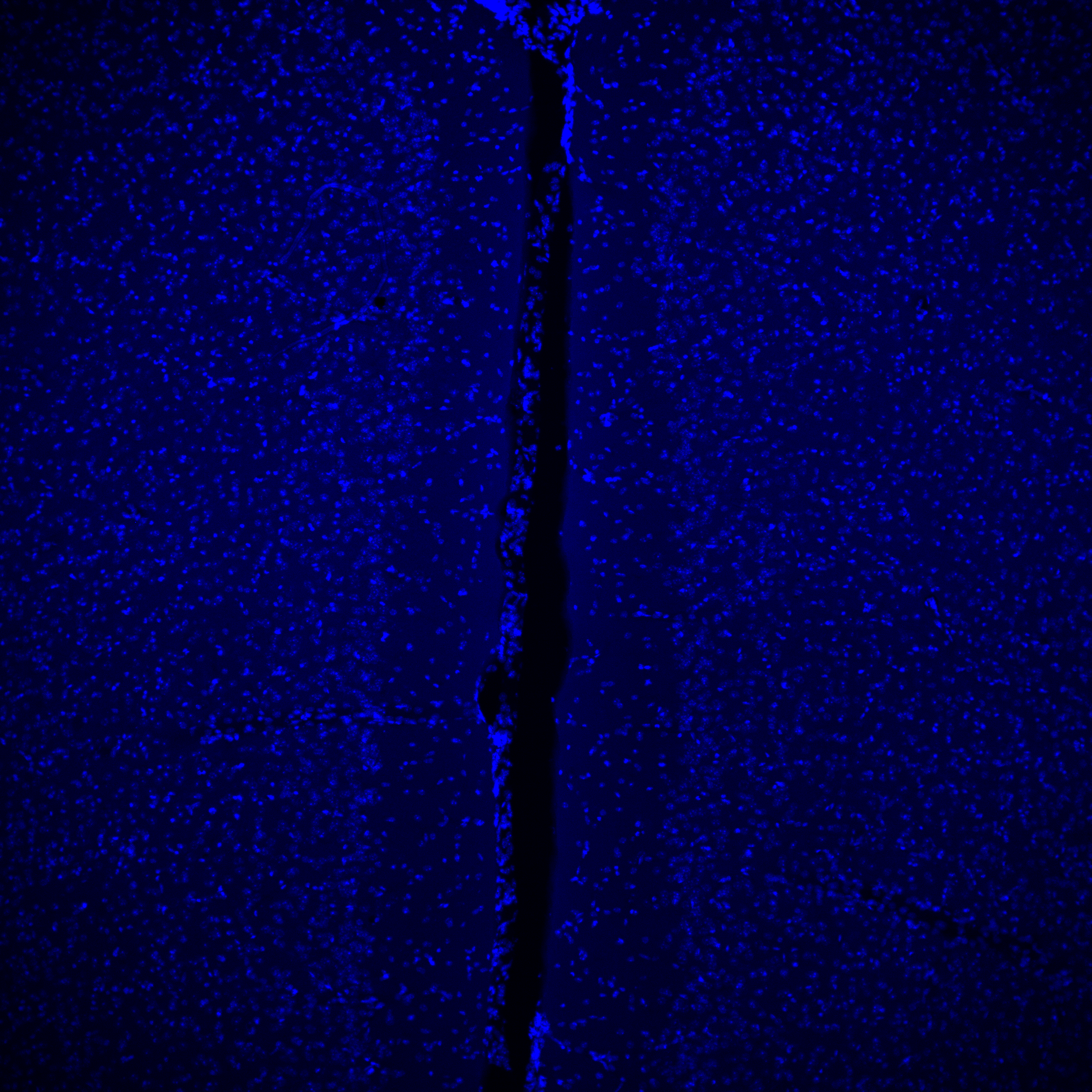

Supplement: Supplementary file 3 — Source data Fig. 1 [file 44321_2024_179_MOESM3_ESM.zip › 1G-20241022T063913Z-001/1G/SPF/1_DAPI.png]

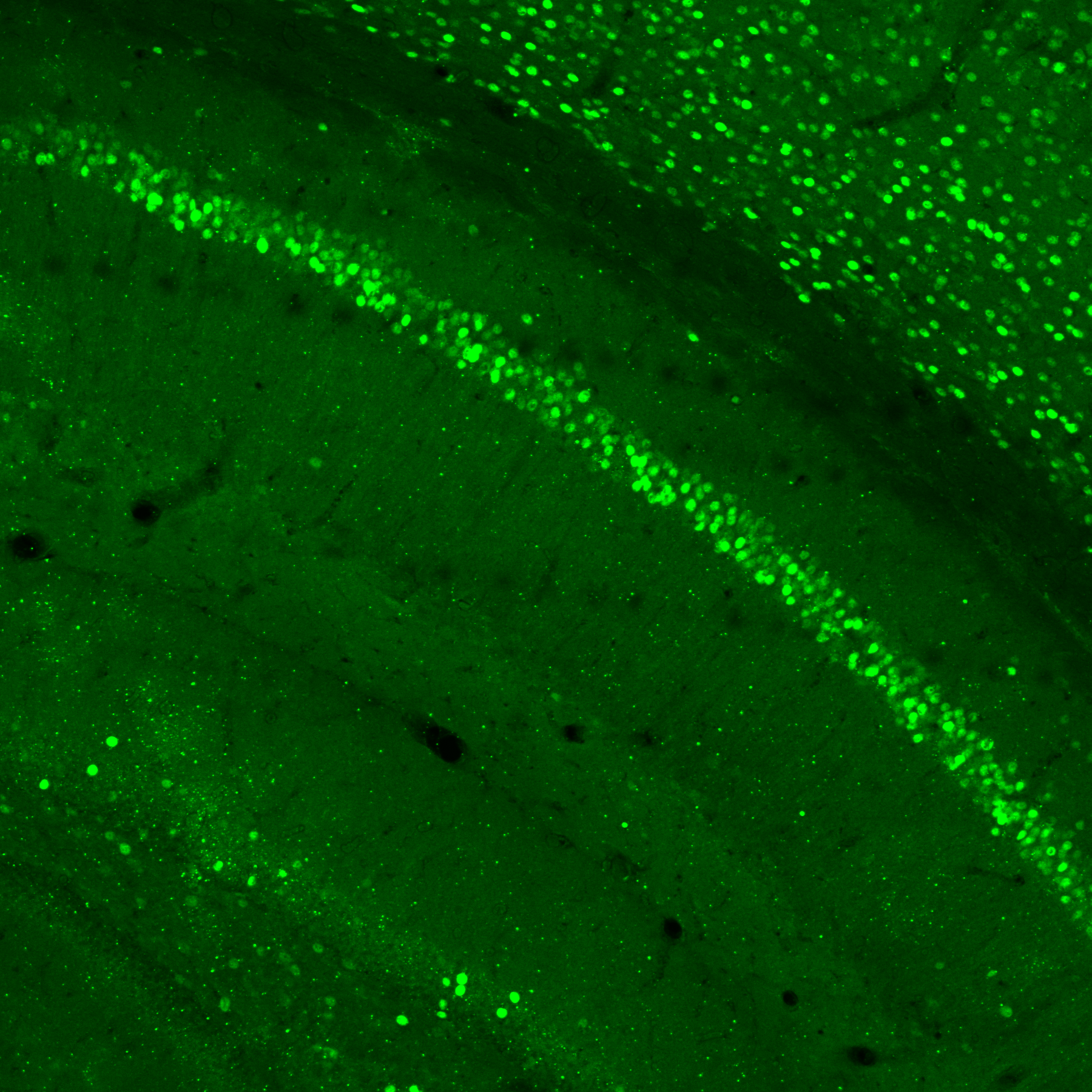

Supplement: Supplementary file 3 — Source data Fig. 1 [file 44321_2024_179_MOESM3_ESM.zip › 1H-20241022T063636Z-001/1H/GF/1212.png]

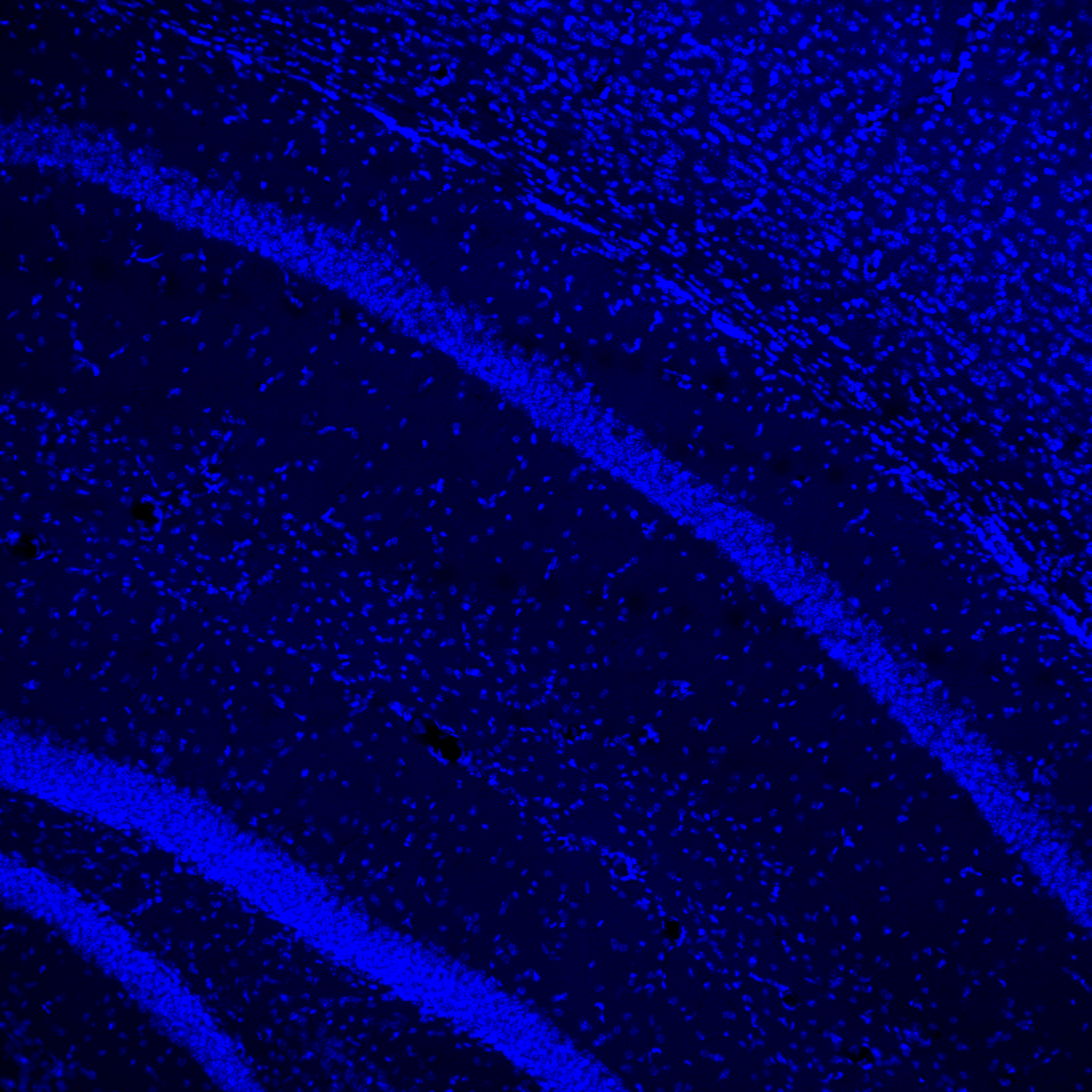

Supplement: Supplementary file 3 — Source data Fig. 1 [file 44321_2024_179_MOESM3_ESM.zip › 1H-20241022T063636Z-001/1H/GF/1212DAPI.png]

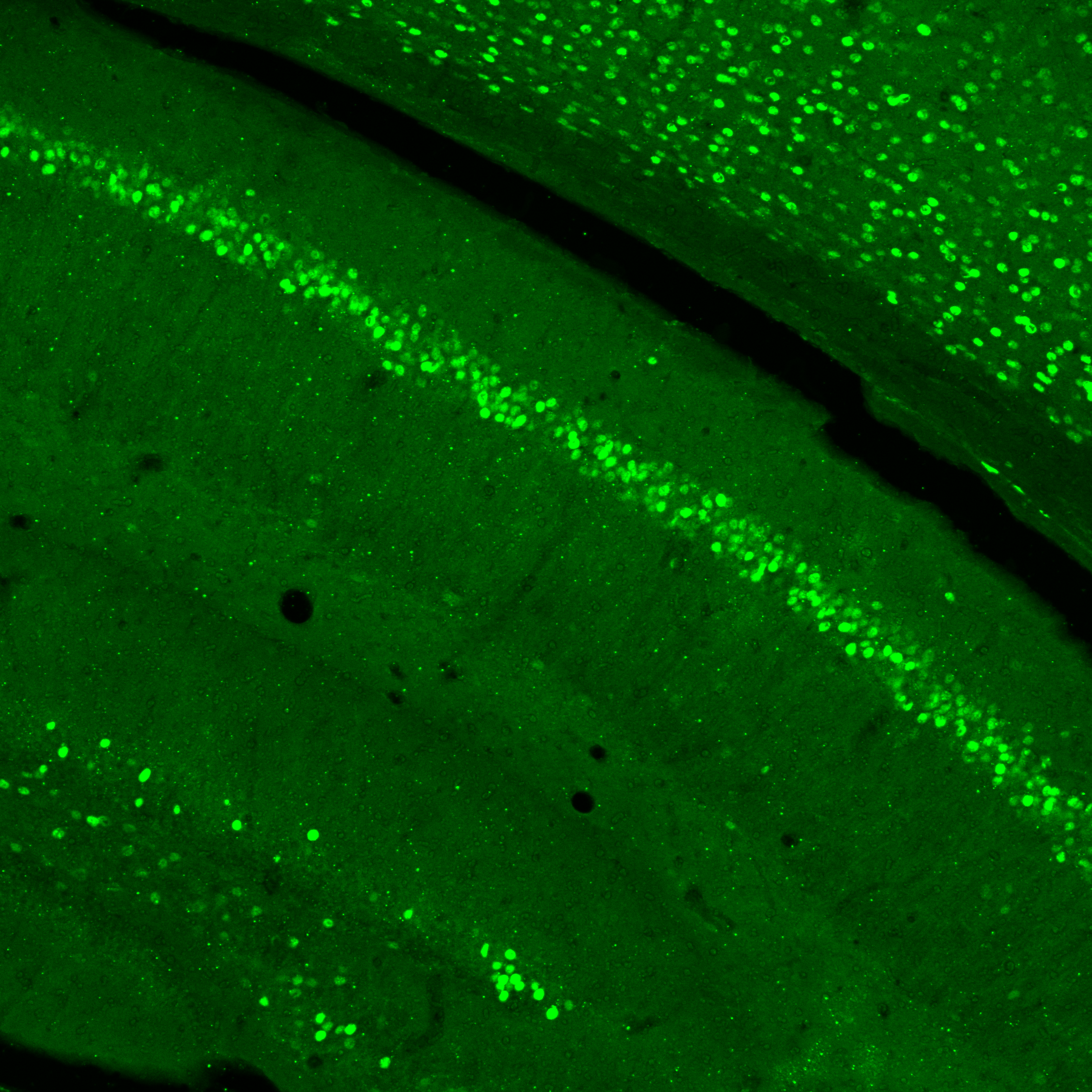

Supplement: Supplementary file 3 — Source data Fig. 1 [file 44321_2024_179_MOESM3_ESM.zip › 1H-20241022T063636Z-001/1H/SPF/[회전]1219.png]

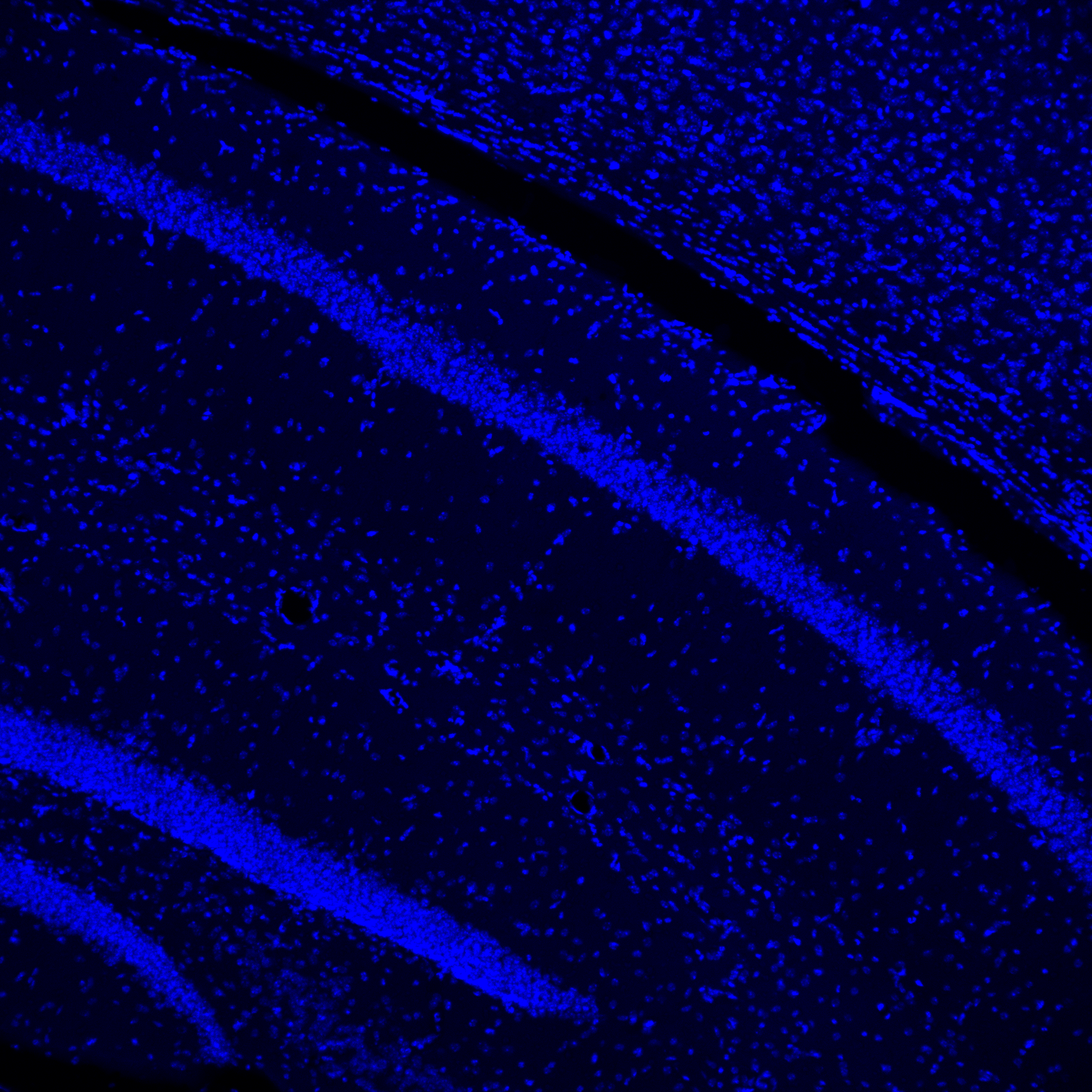

Supplement: Supplementary file 3 — Source data Fig. 1 [file 44321_2024_179_MOESM3_ESM.zip › 1H-20241022T063636Z-001/1H/SPF/[회전]1219DAPI.png]
